# Extracorporeal cardiopulmonary resuscitation in adults and children: A review of literature, published guidelines and pediatric single-center program building experience

**DOI:** 10.3389/fmed.2022.935424

**Published:** 2022-11-21

**Authors:** Taylor Olson, Marc Anders, Cole Burgman, Adam Stephens, Patricia Bastero

**Affiliations:** ^1^Pediatric Critical Care Medicine, Children's National Hospital, Washington, DC, United States; ^2^Department of Pediatrics, Baylor College of Medicine, Houston, TX, United States; ^3^Pediatric Critical Care Medicine, Texas Children's Hospital, Houston, TX, United States; ^4^ECMO, Texas Children's Hospital, Houston, TX, United States; ^5^Department of Surgery, Baylor College of Medicine, Houston, TX, United States; ^6^Congenital Heart Surgery, Texas Children's Hospital, Houston, TX, United States

**Keywords:** ECMO—extracorporeal membrane oxygenation, ECPR program, ECPR training, extracorporeal resuscitation, pediatric ECPR, adult ECPR

## Abstract

Extracorporeal cardiopulmonary resuscitation (ECPR) is an adjunct supportive therapy to conventional cardiopulmonary resuscitation (CCPR) employing veno-arterial extracorporeal membrane oxygenation (VA-ECMO) in the setting of refractory cardiac arrest. Its use has seen a significant increase in the past decade, providing hope for good functional recovery to patients with cardiac arrest refractory to conventional resuscitation maneuvers. This review paper aims to summarize key findings from the ECPR literature available to date as well as the recommendations for ECPR set forth by leading national and international resuscitation societies. Additionally, we describe the successful pediatric ECPR program at Texas Children's Hospital, highlighting the logistical, technical and educational features of the program.

## Introduction

Extracorporeal cardiopulmonary resuscitation (ECPR) as an adjunct to conventional cardiopulmonary resuscitation (CCPR) employs veno-arterial extracorporeal membrane oxygenation (ECMO) in the setting of refractory cardiac arrest. CCPR provides 25–30% of normal cardiac output, while extracorporeal perfusion techniques provide optimized circulatory support and end organ perfusion (1–3). The utilization of ECPR has grown dramatically over the last two decades in both adult and pediatric populations (4–9). The Extracorporeal Life Support Organization (ELSO) is an international non-profit consortium of health care institutions, researchers, and industry partners that maintains a data registry of ECMO patients from more than 450 centers. More than 154,000 patients have been reported to ELSO, with ECPR cases representing 12% of ECMO cases. Of the 18,000 ECPR cases, 12% are neonatal, 31% pediatric, and 57% adult, representing 5, 18, and 13% of all neonatal, pediatric, and adult ECMO cases respectively (4). The uptake and expansion of ECPR has been exponential, with fewer than 100 adult cases reported to ELSO per year before 2009 and more than 1,500 cases per year after 2018 (4).

ECPR is a resource-intensive resuscitation modality with logistical and technical challenges, availability limitations and an unclear economic impact. However, there is growing evidence suggesting potential benefit on patient outcomes. This review paper aims to summarize key findings from the ECPR literature available to date as well as the recommendations for ECPR set forth by national and international leading resuscitation societies. Additionally, we describe the successful pediatric ECPR program at Texas Children's Hospital, highlighting the logistical, technical and educational features of the program.

## Adult ECPR

### Epidemiology

In the United States, the annual incidence of adult in-hospital cardiac arrests (IHCA) is an estimated 292,000 per year with 12–26% survival (10–15). Additionally, there are nearly 350,000 adult out-of-hospital cardiac arrests (OHCA) per year, accounting for one in five deaths in the United States (15–17). OHCA survival ranges from 8 to 15% and is influenced by a multitude of pre-hospital factors including arrest location, bystander presence, cardiopulmonary resuscitation (CPR) quality, etiology of cardiac arrest, initial cardiac rhythm, emergency medical services response time, time to defibrillation, time to return of spontaneous circulation (ROSC), as well as post-resuscitation care (11, 15, 17–25).

Despite advancements in resuscitation science, outcomes for adult IHCA and OHCA remain poor. Attempts to improve these outcomes, coupled with broadened availability and increased uptake of ECMO technology have resulted in the development of ECPR for refractory cardiac arrest. The use of ECPR is growing, with a tenfold increase from 2003 to 2014 and more than 10,000 adult ECPR cases reported to ELSO with a contemporary survival rate of 30% (4, 26).

### Adult extracorporeal cardiopulmonary resuscitation

Naturally, the ECPR literature has investigated superiority over CCPR, including several meta-analyses as well as smaller, retrospective and prospective observational studies. Overall, these data support the use of ECPR, with largely positive reported effects on survival and neurologic outcome (27–35). For example, the meta-analysis by Ouweneel et al. (29) analyzed over 3,000 IHCA and OHCA patients receiving ECPR and CCPR and found an increase in 30-day survival and favorable neurologic outcome with ECPR, findings which were upheld on propensity analysis of over 400 patients, adjusting for likelihood of receiving ECPR. However, there are inherent challenges in comparing these modalities in a non-controlled manner with high risk of bias due to fundamental group differences in type of arrest and patient comorbidities, as well as potential for heterogeneity across studies. Study heterogeneity was cited as the primary reason precluding meta-analysis in the work performed by Holmberg et al. (36) for the International Liaison Committee on Resuscitation (ILCOR) 2019 consensus statement.

Despite its growing use, ECPR is employed in <1% of adult IHCA, with use influenced by patient age, comorbidities, cardiac diagnoses and time and location of arrest (37). ECPR for adult IHCA is investigated by multiple studies (6, 27, 30, 38–68). Meta-analyses reveal promising pooled survival rates ranging from 30 to 38%, with 84% of survivors achieving favorable neurologic outcome (7, 69). However, meta-analyses comparing ECPR to CCPR in IHCA alone demonstrate mixed results, with improved ECPR outcomes noted by Chen et al. (30) but no significant difference reported by Wang et al. (27).

As a result of evolving technology and deployment efficiency, ECPR is utilized more frequently in OHCA settings as well (31, 35, 48, 66, 67, 70–99). A recent, large retrospective study by Bougouin et al. (84) analyzed 13,000 cases of OHCA from 2011 to 2018 in the Paris region, with 4% of patients receiving ECPR, and demonstrated no survival benefit for ECPR (8%) compared to CCPR (9%). However, several single center prospective and retrospective studies demonstrated improved survival with ECPR up to 43% (48, 72, 82, 83, 88, 96, 97). Similarly, a recent meta-analysis by Downing et al. of 44 studies inclusive of 3,097 patients identified an ECPR survival rate of 24%, with 18% of all patients having a good neurological outcome, consistent with previous meta-analyses (31, 35).

Currently there are only two published randomized controlled trials directly comparing ECPR and CCPR for OHCA, the “Advanced reperfusion strategies for patients with out-of-hospital cardiac arrest and refractory ventricular fibrillation (ARREST)” trial and “Effect of Intra-arrest Transport, Extracorporeal Cardiopulmonary Resuscitation, and Immediate Invasive Assessment and Treatment on Functional Neurologic Outcome in Refractory Out-of-Hospital Cardiac Arrest: A Randomized Clinical Trial” (8, 9). As for the ARREST trial, the authors report 43% survival to hospital discharge in patients treated with ECPR compared to 7% with CCPR. After enrollment of only 30 patients, the study was terminated at the first interim analysis given that the posterior probability of ECMO superiority exceeded the prespecified monitoring boundary. Of note, all patients in the ARREST trial had an initial shockable rhythm and benefited from a streamlined process for ECPR initiation (8). The other randomized trial by Belohlavek et al. evaluated 256 OHCA patients, including all presenting rhythms (ventricular fibrillation, asystole and pulseless electrical activity), and reported 32% survival with good neurological outcome at 180 days in the ECPR group compared to 22% in the CCPR group (*p* = 0.09) (9). Of note, the trial was stopped prematurely when prespecified criteria for futility were met, however, the authors note that the trial was possibly underpowered to detect a clinically relevant difference (9).

Available data suggest that ECPR survival and neurologic outcome are impacted by various clinical and patient specifics. Favorable predictors include age (43, 68, 99), IHCA (48, 51, 67, 100, 101), shockable rhythm (28, 55, 68, 78, 84, 97–99, 102, 103), temporary ROSC (68, 84, 104), witnessed arrest with bystander CPR (104), shorter CCPR duration or time from arrest to ECMO (28, 43, 51, 64, 67, 68, 83, 86, 87, 94, 96, 102–104), higher baseline pH (93, 98, 99, 102, 103), lower baseline lactate (51, 86, 98, 102, 103) and percutaneous coronary intervention (43, 48, 91, 102). Pre-hospital cannulation for OHCA ECPR may have additional benefits on survival and neurologic outcome by reducing low-flow time for patients with longer anticipated transport times (83, 84, 91, 95, 105).

In light of the available literature, leading national and international resuscitation societies have set forth guidelines on the use of ECPR in adults. Table 1 summarizes the recommendations from the American Heart Association (AHA), European Resuscitation Council (ERC), ILCOR (106, 108, 110), and ELSO (114, 115). Of note, ILCOR commissioned a systematic review of ECPR in 2018 which informed the published 2019 Consensus on Science with Treatment Recommendations (CoSTR) (36, 111, 112).

**Table 1:** Published society guidelines on the use of ECPR in adults and children.

	**Adults**	**Children**	
		**IHCA**	**OHCA**
**AHA**	“There is insufficient evidence to recommend the routine use of extracorporeal CPR (ECPR) for patients with cardiac arrest. ECPR may be considered for select cardiac arrest patients for whom the suspected cause of the cardiac arrest is potentially reversible during a limited period of mechanical circulatory support.” (106) Class of recommendation: 2b Weak Level of evidence: Limited Data (C-LD)	Cardiac: “ECPR may be considered for pediatric patients with cardiac diagnoses who have IHCA in settings with existing ECMO protocols, expertise, and equipment.” (107) Class of recommendation: 2b Weak Level of evidence: Limited Data (C-LD) Non-cardiac: “There is insufficient evidence to suggest for or against the use of ECPR for….pediatric patients with noncardiac disease experiencing IHCA refractory to conventional CPR.” (107)	“There is insufficient evidence to suggest for or against the use of ECPR for pediatric patients experiencing OHCA…” (107)
**ERC**	“Consider extracorporeal CPR (eCPR) as a rescue therapy for selected patients with cardiac arrest when conventional ALS measures are failing or to facilitate specific interventions (e.g., coronary angiography and percutaneous coronary intervention (PCI), pulmonary thrombectomy for massive pulmonary embolism, rewarming after hypothermic cardiac arrest) in settings in which it can be implemented.” (108)	“…We advise considering eCPR for children with ED-or IHCA with a presumed or confirmed reversible cause where conventional ALS does not promptly lead to ROSC (weak recommendation, very low certainty evidence). An essential precondition is the organizational setting i.e., with a strong institution-based commitment to sustaining a resuscitation system that includes eCPR with appropriate quality improvement systems. To make a realistic choice about the use of eCPR, systems should also consider the evidence on cost-efficiency…” “Should ECPR be considered as a rescue therapy for septic IHCA the ECMO team must be activated early after initiation of PALS based on institution-specific protocols” (109)	“Given the high resources needed and the fact that outcome is related to time to initiation and quality of CPR before initiation, the indications for eCPR in OHCA are very limited.” (109) Appendix RR 33.3 expands, “the writing group would consider E-CPR for OHCA in case (1) it concerns a deep hypothermic arrest …and/or (2) cannulation can be done prehospitally by a highly trained team, within a dedicated healthcare system that accounts for this (provided the no flow + low flow time is known and limited and the cause truly reversible).” (109)
**ILCOR**	“…ECPR may be considered as a rescue therapy for selected patients with cardiac arrest when conventional CPR is failing in settings in which it can be implemented (weak recommendation, very low-certainty evidence).” (110–112)	“…ECPR may be considered as an intervention for selected infants and children (eg, pediatric cardiac populations) with IHCA refractory to conventional CPR in settings where resuscitation systems allow ECPR to be well performed and implemented (weak recommendation, very low-quality evidence)” (111–113)	“There is insufficient evidence in pediatric OHCA to formulate a treatment recommendation for the use of ECPR.” (111–113)
**ELSO**	“We recommend that institutions offering ECPR develop a guideline for ECPR treatment which includes eligibility, goals of treatment, and a timeline with conditions for stopping ECMO in those without neurologic recovery, or in those ineligible for long-term mechanical cardiac support because of insufficient cardiac recovery.” (114)	“We suggest that institutions establish local protocols that guide their use of conventional CPR with or without ECPR. If institutions opt to deploy protocols that involve ECPR, one of the early steps of this protocol must include decision making by a senior clinician based on physiologic principles. Combining high-quality ECPR with high-quality conventional CPR may be considered if the cardiopulmonary arrest is witnessed and is associated with a reversible condition. Unwitnessed events in all settings have a poor prognosis and should be considered a relative contraindication for ECPR.” (115)	“In children, there are insufficient data to support the recommendation for the use of ECPR for out-of-hospital cardiopulmonary arrest events, either applied in the field (e.g., trauma or remote retrievals of avalanche or drowning victims) or in the hospital after ongoing conventional CPR during transport.” (115)

IHCA, in-hospital cardiac arrest; OHCA, out-of-hospital cardiac arrest; AHA, American Heart Association; ERC, European Resuscitation Council; ILCOR, International Liaison Committee On Resuscitation; ELSO, Extracorporeal Life Support Organization; CPR, cardiopulmonary resuscitation; ECPR, extracorporeal cardiopulmonary resuscitation; ECMO, extracorporeal membrane oxygenation; ALS, advanced life support; ED, emergency department; ROSC, return of spontaneous circulation; PALS, pediatric advanced life support.

### ECPR candidacy

ECPR patient selection is a crucial determination as it relates to ECPR program building as well as research trial design. Data that definitively identify the patients most likely to benefit from ECPR are still needed, and thus candidacy determinations will be ECPR center or trial dependent. In general, ECPR is thought to provide a bridge to process reversal (e.g., myocardial infarction requiring percutaneous coronary intervention), other definitive management (e.g., ventricular assist device or organ transplantation), or further information seeking (106, 108, 114). Informed, patient-tailored institutional decisions about ECPR candidacy should occur as early as possible (114). The 2021 ELSO Interim Guideline Consensus Statement on ECPR in adults proposes the following inclusion criteria: age <70 years, witnessed arrest, arrest to CPR time <5 min, initial rhythm ventricular fibrillation, ventricular tachycardia, or pulseless electrical activity, arrest to ECMO time <60 min, end tidal carbon dioxide >10 mmHg during CCPR prior to cannulation, intermittent ROSC or recurrent ventricular fibrillation, “signs of life” during CCPR, absence of greater than mild aortic valve incompetence, and absence of significant end-stage organ failures or comorbidities (114). However, the majority of contemporary ECPR programs do not have formalized inclusion and exclusion criteria (116).

### Post-ECPR care

Mortality after ROSC can be at least partially attributed to the complex pathophysiologic state following cardiac arrest involving the initial ischemic insult as well as reperfusion and post-reperfusion injuries and their effect on organ systems, referred to as “post-cardiac arrest syndrome” (117). Post-resuscitation care is a resource-intensive multidisciplinary undertaking that requires critical monitoring and tailored management strategies to prevent additional injury, as well as investigation of the precipitating cause and reversal or treatment if necessary.

Recommendations for post-resuscitation care are set forth by AHA, informed most recently by an ILCOR 2020 systematic review and CoSTR for advanced life support (106, 110). These recommendations highlight avoidance of hypotension using crystalloid, inotropes, or mechanical support as needed (goal systolic blood pressure ≥90 mmHg; mean arterial blood pressure ≥65 mmHg), avoidance of hypoxemia and hyperoxemia (target oxygen saturation of 92–98%), and maintenance of normocapnia (arterial partial pressure of carbon dioxide 35–45 mmHg) (106, 110). Other components of care include maintenance of euglycemia and timely diagnosis and treatment of seizures. Prophylactic use of anti-seizure medications, antibiotics and steroids are not advised (106, 110). Lastly, while hyperthermia prevention and management are universally accepted, targeted temperature management (TTM) is an area of ongoing research and relative controversy. TTM was recommended between 32 and 36°C for at least 24 h for comatose adults following ROSC after IHCA or OHCA with any initial rhythm, based on landmark studies by Bernard et al. and Hypothermia after Cardiac Arrest Study Group (118, 119). However, a recent large, open-label trial comparing targeted hypothermia and normothermia found no difference in 6-month survival or functional outcome following OHCA (120). In light of this and other evidence, the ERC revised their guidelines on temperature control, advising continuous temperature monitoring and preventing fever (>37.7°C) for 72 h, but there was insufficient evidence to recommend for or against temperature control at 32–36°C (121).

While additional data is needed to inform best practices for post-ECPR care, providers should consider general post-resuscitation care recommendations as well as the ELSO Interim Guideline Consensus Statement on ECPR in adults (114). After ECMO blood flow of 3–4 L/min is achieved, providers should target mean arterial blood pressure ≥60 and <80 mmHg (114). Unintended hyperoxia and hypocapnia should be avoided, as they have been associated with worse outcomes for patients receiving ECPR (122, 123). Prompt and frequent measurements of oxygenation and ventilation are needed, with adjustments to ECMO oxygen gas and sweep flow to target arterial oxygen saturation of 92–97% and normocarbia (114). With respect to temperature control, the circuit warmer can be programmed as desired, and early intervention for hyperthermia is similarly feasible using the circuit warmer and heat exchanger. Lastly, patients exhibiting signs of left ventricular hypertension and pulmonary edema should be considered for left ventricular unloading and/or venting (114).

## Pediatric ECPR

### Epidemiology

There are over 15,000 pediatric in-hospital cardiac arrests requiring CPR per year in the United States, with several studies documenting an increase in survival over time (10, 11, 124, 125). Pediatric IHCA occurs in 2–6% of intensive care admissions, and the majority of arrests occur in intensively monitored patients (90%) with a secured airway (80%) (11, 126–128). Survival rates for pediatric IHCA are 40–49% with 34–90% of survivors achieving favorable neurological outcome depending on the study (11, 15, 128, 129). Additionally, over 5,000 children experience non-traumatic OHCA annually (130). In contrast to adult OHCA, pediatric OHCA is more commonly attributed to non-cardiac etiologies (131). Survival rates are ~11–13%, with favorable neurological outcomes occurring in 85–90% of survivors (11, 15).

### Pediatric extracorporeal cardiopulmonary resuscitation

Pediatric ECPR, like adult ECPR, aims to optimize cardiac output and end organ perfusion with the goal of improving arrest survivability and neurological outcomes. The most recent ELSO Registry International Summary Report, comprising data from inception through 2020, identifies 5,682 pediatric ECPR cases (4). ECPR cases account for 18% of all pediatric ECMO cases, which has markedly increased from 5% in 2004 (4, 132). The survival rate to discharge for pediatric ECPR is 42% per the ELSO Registry, with over 70% of cases performed for an indication of cardiac disease (4, 133). Similarly, a recent systemic review and meta-analysis of 28 studies and 1,300 pediatric ECPR patients (excluding registry patients) demonstrated 30% survival with favorable neurological outcome (134). A secondary analysis of the IHCA “Therapeutic Hypothermia After Pediatric Cardiac Arrest” (THAPCA) trial demonstrated that ECPR survivors had similar long term neurobehavioral outcomes to other post-arrest survivors, with one third of ECPR patients alive with good functional status at 1 year (135, 136). Despite the steady growth of ECPR utilization over time in pediatric patients, the pediatric ECPR literature lacks randomized studies and the majority of data is focused on IHCA in cardiac patients (4, 5, 134, 137–149).

In a landmark paper, Lasa et al. performed a large systematic comparison of ECPR and CCPR for pediatric patients with IHCA requiring >10 min resuscitation using the Get With the Guidelines-Resuscitation Registry (GWTG-R), a large multicenter national registry. Their analysis demonstrated improved survival and survival with favorable neurological outcome for the ECPR group compared to the CCPR group even after adjusting for illness type, pre-existing conditions, and arrest features. Their findings were further confirmed on propensity score-matched analysis (150).

Analyses of the GWTG-R and the National Registry of Cardiopulmonary Resuscitation demonstrated use of ECPR in 3–7% of IHCA, with the most frequent utilization for cardiac surgical patients (150–153). In fact, ECPR was used in 27% of arrests between 2014 and 2016 according to the Pediatric Cardiac Critical Care Consortium registry (154). Use of ECPR for refractory IHCA has increased threefold in the last two decades according to the National Inpatient Sample dataset (128). Overall, registry analyses show ECPR survival is 27–44% for IHCA with neurologically favorable outcomes in 56–73% of survivors (150–152, 155). Several studies demonstrated improved ECPR survival (32–48%) for patients with cardiac disease compared to non-cardiac patients (151, 152, 156–159). A meta-analysis including 762 patients by Joffe et al. (155) described overall ECPR survival of 49%, with favorable neurological outcomes occurring in 79% of survivors and non-cardiac disease associated with increased risk of mortality. Other retrospective single, multicenter, and database studies describe 25–73% overall survival and 50–100% of survivors having good neurologic outcome (137, 145, 147, 149, 156, 159–176).

The literature is scarce regarding the use of ECPR for pediatric OHCA, which comprises only 3% of ECPR cases reported to ELSO (138). From 2010 to 2019, 33 patients (7 survivors) reported to ELSO experienced arrest during emergency medical services transport and received ECPR by the receiving institution (Bilodeau and McMullan, personal communication). ECPR in pediatric patients reported to ELSO Registry (Unpublished raw data). Additionally, ECPR for pediatric OHCA is described by infrequent single center reports (177, 178). Likewise, there is only one case report of out-of-hospital cannulation in a pediatric patient (179).

Aside from preexisting non-cardiac disease, patient factors associated with ECPR outcomes include pre-ECPR pH, lactate and renal injury as well as post-ECPR lactate and pH (133, 135, 146, 147, 149–151, 157–160, 163, 164, 168, 171–173, 175, 180, 181). In a secondary analysis of ECPR within the IHCA THAPCA trial, receipt of open-chest cardiac massage was associated with shorter CPR duration, survival and good neurological outcome at 1 year, while open-chest cardiac massage is a negative predictor in other studies (135, 164). Increasing CPR interruptions during cannulation have been associated with worse outcomes (145). Additionally, several studies have described an association between CPR duration and survival (145, 147–149, 158, 160, 162, 164, 165, 168, 172, 174, 175). Meanwhile, other studies have not upheld this finding, and moreover, some authors have recognized the ability of ECPR to rescue patients with very prolonged arrest times such as 60–95 min (146, 156, 158, 166, 169–171, 180). Arrest rhythm has also been inconsistently associated with outcomes (124, 135, 149, 158, 182). Complications while on ECMO portend a worse prognosis (133, 157–159, 168, 171, 173).

In light of the available literature, leading national and international resuscitation societies have set forth guidelines on the use of ECPR in children. Table 1 summarizes the recommendations from AHA, ERC, ILCOR and ELSO (107, 109, 113, 115).

### ECPR candidacy

Data are currently insufficient to definitively identify appropriate pediatric ECPR candidates. A recent survey of pediatric critical care physicians demonstrated that ECPR activation decisions are complex and heterogeneous, but most consistently influenced by patient diagnosis (cardiac vs. non-cardiac), CPR duration, arrest location, witnessed arrest, and blood pH (183). ECPR consideration is currently advised by AHA for patients with cardiac diagnoses when CCPR is failing, but should be reserved to experienced institutions (107). There are insufficient data to advise uniform application in other pediatric subgroups (107).

### Post-ECPR care

Following the application of ECPR, optimal post-resuscitation care for children is critical. Post-cardiac arrest syndrome in children mimics that in adults, involving brain injury, myocardial dysfunction, systemic ischemia and reperfusion injury, and unresolved inciting pathology (184). AHA recommendations for pediatric post-resuscitation care include optimizing hemodynamics (systolic blood pressure >5th percentile for age) utilizing crystalloid and/or vasopressor agents as needed, targeting normoxemia (oxygen saturations 94–99%) and limiting exposure to severe hypercapnia or hypocapnia. Oxygenation and ventilation targets may be modified in the context of a patient's underlying condition (107, 184). With respect to TTM following pediatric cardiac arrest, the AHA recommends continuous measurement of core temperature and targeting either normothermia (36–37.5°C) for 5 days or hypothermia (32–34°C) for 2 days followed by normothermia (36–37.5°C) for 3 days (107, 184, 185). Evaluated robustly by the multicenter randomized controlled THAPCA study, TTM at 32–34°C when compared to TTM at 36–37.5°C had no effect on survival or neurologic outcome (136, 186). Additionally, hyperthermia is common, and if persistent, is associated with worse neurological outcomes and should therefore be aggressively treated (107, 184, 187).

While there is no literature specific to post-ECPR care in children, management should at minimum uphold the recommendations for general post-resuscitation care. As is true for adults upon ECPR initiation, ECMO circuit flows in conjunction with fluids and vasopressors should be titrated to optimize hemodynamics. Extracorporeal support has the potential to mitigate post-arrest hypotension which is common, and associated with worse outcomes (188). In addition, institution of extracorporeal support should prompt attention to oxygenation (risk of hyperoxia), ventilation and ECMO sweep gas flow (risk for hypocapnia), and desired temperature management (programmed temperature control with circuit warmer and heat exchanger).

## Neonatal ECPR

There are 2,261 cases of neonatal ECPR reported to the ELSO Registry with an overall survival to discharge of 42% (4). Approximately half of ELSO neonatal ECMO centers report performing neonatal ECPR (189). Neonatal cases are included within GWTG-R studies, representing 21% of pediatric ECPR cases with 38% survival and 46% of survivors having favorable neurological outcome (150). Post-cardiotomy ECPR cases for neonates with congenital heart disease predominate (181, 190, 191). Additionally, neonates may require ECPR in the setting of respiratory disease, which carries a favorable prognosis (133). Other positive prognostic indicators identified by the literature include greater gestational age, weight, and cardiac arrhythmia causing arrest (190). Unfavorable factors include lower pre-ECPR arterial oxygenation, delayed lactate clearance, ECMO duration and complications (181, 190).

## ECPR program building

ECPR is a complex multi-disciplinary resuscitation modality that involves substantial resources and strong institutional commitment. Successful ECPR programs require maintenance of expertise and equipment, as well as integrated quality improvement mechanisms to assess important program metrics (114).

**Figure 1:**
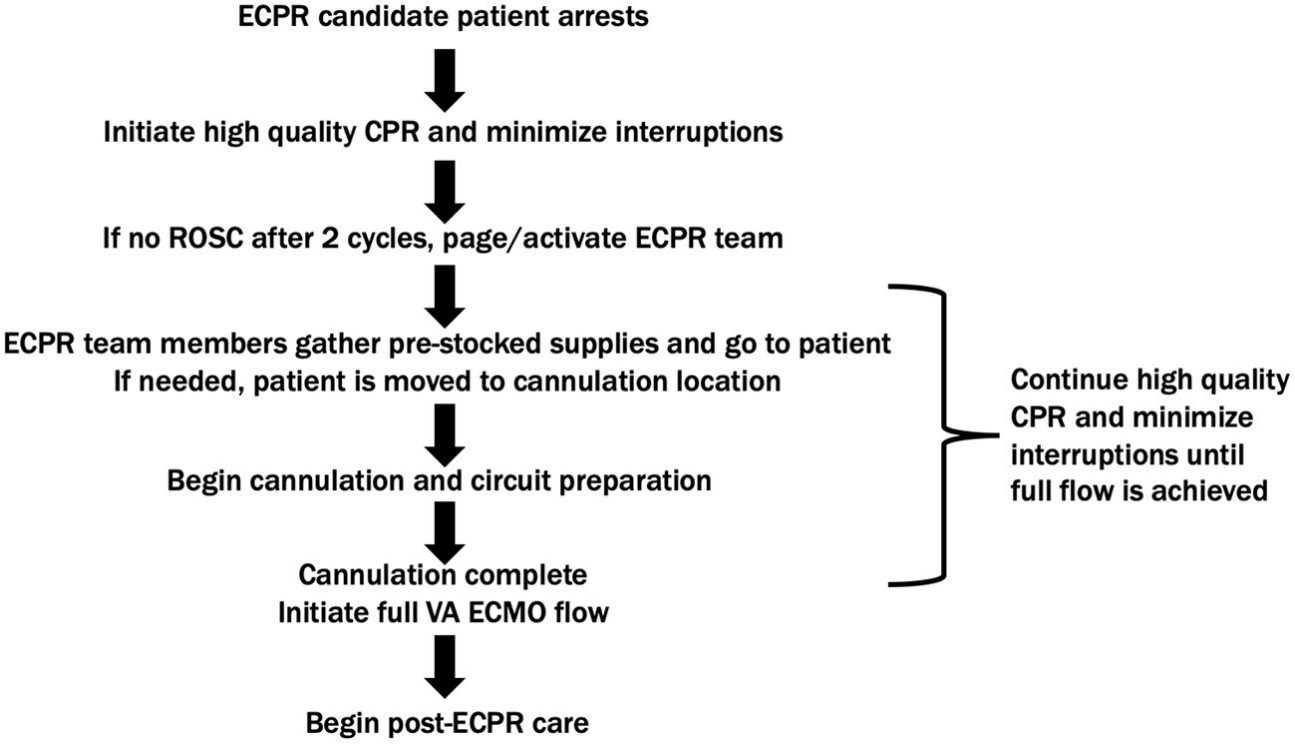
ECPR deployment algorithm. ECPR, extracorporeal cardiopulmonary resuscitation; CPR, cardiopulmonary resuscitation; ROSC, return of spontaneous circulation; VA ECMO, veno-arterial extracorporeal membrane oxygenation.

A successful ECPR program has been established at Texas Children's Hospital since 2005. Figure 1 details an example ECPR deployment algorithm. ECPR candidacy is discussed preemptively in high-risk patients. All cardiac patients are ECPR candidates, except for those with significant comorbidities or vascular access limitations. Certain non-cardiac patients are considered on a case-by-case basis, including but not limited to patients with pulmonary hypertension, drug overdose, and pulmonary embolism. Candidate patients experiencing a witnessed arrest in pre-defined locations may receive ECPR, including the emergency department (ED), intensive care units (ICU), cardiac catheterization lab, or operating room (OR). Activation can be triggered by either cardiac intensive care physician or congenital heart surgeon, when high-quality CPR fails to achieve sustainable ROSC after two cycles. A staff member calls in to the operator requesting ECPR activation and system page out, which notifies congenital heart surgeon, operating room staff, cardiovascular anesthesia, perfusionists, and pharmacy as to the patient location and weight. The congenital heart surgery attending and fellow are available on-call 24/7. During regular business hours, the team is in-house, however, after-hours, it is expected that they are within a 20-min distance from the hospital should ECPR activation be needed. The assisting operating room team, bedside team, and ECMO technical team are in-house 24/7 and would start preparing before the arrival of the surgical team. Each team member adheres to their protocol that includes equipment gathering. Bundled surgical cannulation carts and crystalloid-primed ECMO circuits are pre-prepared and maintained in key locations dedicated to rapid deployment. ECPR cannulation takes place in specific hospital locations including the OR, cardiac catheterization lab, or ICU. Thus, candidate patients experiencing arrest in the ED will be transported. The optimal ECPR cannulation strategy is dependent upon patient anatomy, size, operative status, and pathophysiology. For example, a post-sternotomy patient with open chest may be cannulated centrally given ease of access, while closed-chest, pre-operative, medical cardiac, or non-cardiac patients are more appropriate for initial peripheral cannulation.

Laussen and Guerguerian (192) review the critical components for establishing and sustaining an ECPR program. They describe four time intervals of importance: cardiac arrest to CCPR (interval 1), start of CCPR to ECPR activation (interval 2), ECPR activation to ECMO flow (interval 3), and ECMO flow to post-resuscitation care (interval 4). Interval 1 should not exceed 1 min in the event of witnessed IHCA. Interval 2 can be minimized by early ECPR activation by designated team members. Interval 3 may be reduced when ECPR occurs in familiar, well-controlled, protocol-driven environments. Many centers utilize preassigned roles, flowcharts and job aides to enhance staff preparedness and decrease deployment time (177, 191, 193–195). Protocolized, rapid response ECMO programs have proven successful in reducing neurologic complications in both ECPR and general ECMO patients (196).

A key quality initiative for our ECPR program is our clinical event debriefings conducted after all ECPR cases. Started in 2014, these debriefs aim to improve team dynamics and patients' outcome. The structured approach investigates the teams' clinical performance, resources, facilities, including process or system problems to improve. Table top simulations and live simulation based system testings are frequently used to optimize any identified paucities.

### Team training and simulation

Simulation has been widely regarded as beneficial for ECMO team training, technical skills, provider confidence, communication and collaboration, improving deployment times and adherence to ECPR protocols (174, 191, 194, 195, 197–205). Moreover, simulation can be used to develop, test, and/or adapt an ECPR protocol (195, 198, 206).

**Figure 2:**
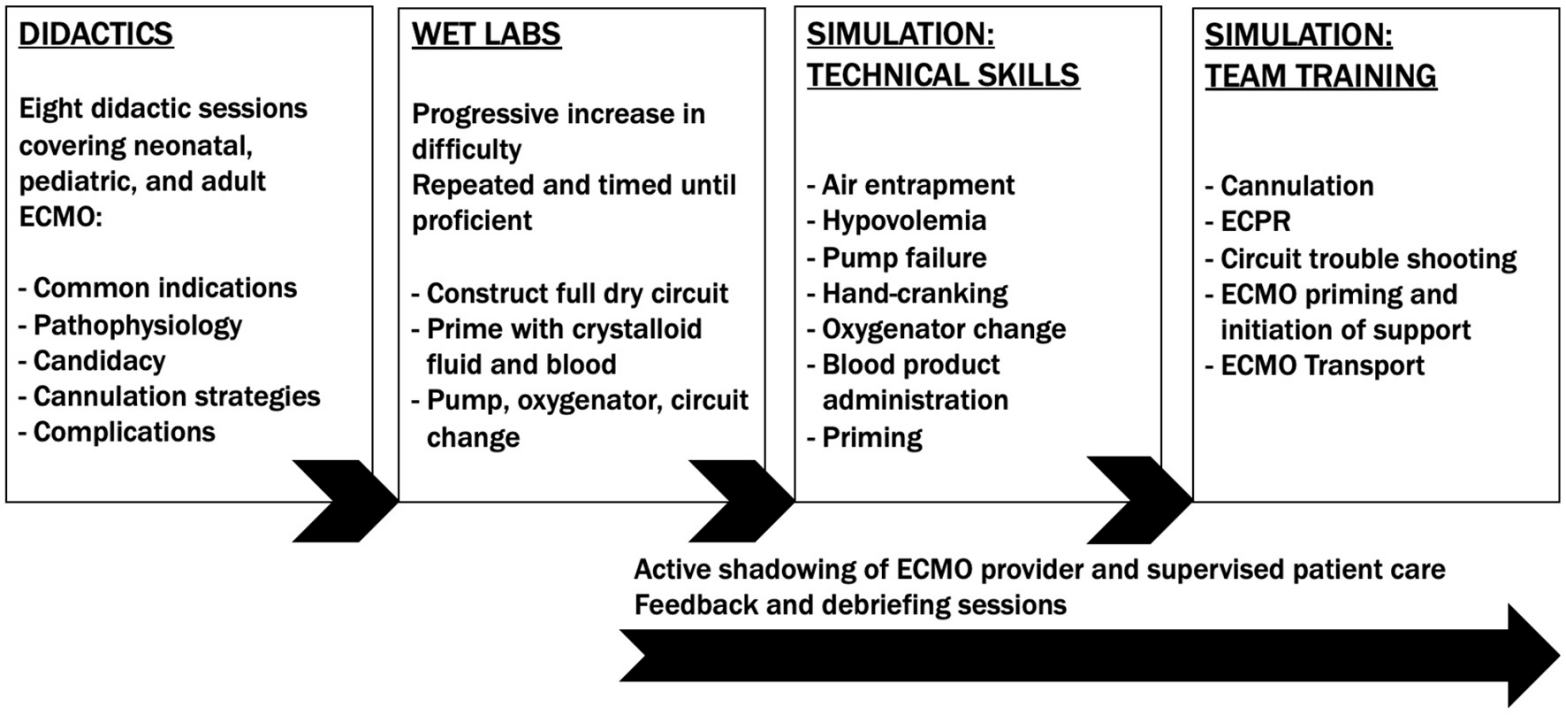
Texas children's hospital ECMO training program. ECMO, extracorporeal membrane oxygenation; ECPR, extracorporeal cardiopulmonary resuscitation.

At Texas Children's Hospital, ECMO team members participate in a structured education and simulation program with competency benchmarks covering all aspects of ECMO delivery (Figure 2). A series of didactic sessions reviews basic fundamentals, including respiratory and cardiac pathophysiology potentially necessitating ECMO support. Didactics further explore ECMO candidacy, cannulation strategies and complications. Subsequently, providers engage in progressively complex wet lab trainings. Wet lab trainings reinforce important processes: setting up a dry circuit (<15 min), priming with crystalloid (<8 min), priming with blood (<5 min), and pump, oxygenator, and circuit exchanges. Wet lab exercises are repeated and timed until proficiency is demonstrated. Next, trainees transition to actively assisting other trained ECMO providers and are evaluated in that capacity. Direct feedback and debriefing sessions provide continuing education.

Multi-disciplinary ECPR simulation sessions are held regularly at Texas Children's Hospital to enhance interprofessional team performance, communication, and technical skills. Scenarios reinforce key components of patient encounters, from deterioration and arrest through ECPR activation, patient transport, cannulation, and circuit management. Additionally, we utilize a surgical simulation model for ECMO cannulation (Image 1; RediStik™ ECMO Cannulation Trainer, Sawbones, Vashon, WA, USA). The model aims to improve procedural skills with ECMO neck cannulation, highly pertinent to ECPR deployment in pediatric patients. Alternate models have been employed by other ECPR simulation programs (199, 207).

## Conclusion

ECPR has the potential to rescue selected patients when conventional resuscitation efforts are failing. There is growing evidence that ECPR improves survival and neurological outcomes in adult OHCA and IHCA. Further well-designed, randomized clinical trials are needed to identify the most appropriate ECPR candidates, as well as optimal pre-ECPR and post-ECPR practices. Additional research is needed to explore the utility of ECPR in pediatric and neonatal populations, with a focus on understanding the role in non-cardiac illness and OHCA. Effective application of ECPR requires substantial health care resources and strong institutional commitment. Institutions should develop a systematic approach, utilizing team training, simulations, and quality improvement mechanisms in order to establish a successful ECPR program.

## Author contributions

PB, MA, and TO contributed to conception, design of the study, wrote the first draft of the manuscript, and edited the final draft to the manuscript submitted. PB, TO, MA, AS, and CB wrote sections of the manuscript. All authors contributed to manuscript revision, read, and approved the submitted version.

## Funding

This work was funded by Michael Pickett MSN, APRN, PNP-BC and Texas Children's Hospital Heart Center Innovation Award 2020, $5k.

## Conflict of interest

The authors declare that the research was conducted in the absence of any commercial or financial relationships that could be construed as a potential conflict of interest.

## Publisher's note

All claims expressed in this article are solely those of the authors and do not necessarily represent those of their affiliated organizations, or those of the publisher, the editors and the reviewers. Any product that may be evaluated in this article, or claim that may be made by its manufacturer, is not guaranteed or endorsed by the publisher.

## Supplementary material

Supplementary Image 1 RediStik™ ECMO Cannulation Trainer. ECMO: extracorporeal membrane oxygenation (https://www.youtube.com/watch?v=UwCJNP94g30&list=PLvWZmgauEkKKcGfI99fYOV9viFyTu03f1&index=2).

## References

[1] Lurie KG Nemergut EC Yannopoulos D Sweeney M. The physiology of cardiopulmonary resuscitation. Anesth Analg. (2016) 122:767–83. 10.1213/ANE.0000000000000926 26562060

[2] Barsan WG Levy RC. Experimental design for study of cardiopulmonary resuscitation in dogs. Ann Emerg Med. (1981) 10:135–7. 7469152 10.1016/s0196-0644(81)80377-0

[3] Kennedy JH. The role of assisted circulation in cardiac resuscitation. JAMA J Am Med Assoc. (1966) 197:615–8. 5953090

[4] Extracorporeal Life Support Organization. ELSO Registry Report International Summary. (2021), 1–40. Available online at: https://www.elso.org/Portals/0/Files/Reports/2021_April/International%20Report%20April_page1.pdf (accessed April 3, 2022).

[5] Nido PJ Dalton HJ Thompson AE Siewers RD. Extracorporeal membrane oxygenator rescue in children during cardiac arrest after cardiac surgery. Circulation. (1992) 86:II300–4. 1424017

[6] Chen YS Lin JW Yu HY Ko WJ Jerng JS Chang WT. Cardiopulmonary resuscitation with assisted extracorporeal life-support vs. conventional cardiopulmonary resuscitation in adults with in-hospital cardiac arrest: an observational study and propensity analysis. Lancet. (2008) 372:554–61. 10.1016/S0140-6736(08)60958-7 18603291

[7] Gravesteijn BY Schluep M Disli M Garkhail P dos Reis Miranda D Stolker RJ. Neurological outcome after extracorporeal cardiopulmonary resuscitation for in-hospital cardiac arrest: a systematic review and meta-analysis. Critical Care. (2020) 24:1–12. 10.1186/s13054-020-03201-0 32807207 PMC7430015

[8] Yannopoulos D Bartos J Raveendran G Walser E Connett J Murray TA. Advanced reperfusion strategies for patients with out-of-hospital cardiac arrest and refractory ventricular fibrillation (ARREST): a phase 2, single centre, open-label, randomised controlled trial. Lancet. (2020) 396:1807–16. 10.1016/S0140-6736(20)32338-2 33197396 PMC7856571

[9] Belohlavek J Smalcova J Rob D Franek O Smid O Pokorna M. Effect of intra-arrest transport, extracorporeal cardiopulmonary resuscitation, and immediate invasive assessment and treatment on functional neurologic outcome in refractory out-of-hospital cardiac arrest: a randomized clinical trial. JAMA. (2022) 327:737–47. 10.1001/jama.2022.6548 35191923 PMC8864504

[10] Holmberg MJ Ross CE Fitzmaurice GM Chan PS Duval-Arnould J Grossestreuer AV. Annual incidence of adult and pediatric in-hospital cardiac arrest in the United States. Circ Cardiovasc Qual Outcomes. (2019) 12:e005580. 10.1161/CIRCOUTCOMES.119.005580 31545574 PMC6758564

[11] Tsao CW Aday AW Almarzooq ZI Alonso A Beaton AZ Bittencourt MS. Heart disease and stroke statistics-−2022 update: a report from the American heart association. Circulation. (2022) 145:e153–639. 10.1161/CIR.0000000000001052 35078371

[12] Ofoma UR Basnet S Berger A Kirchner HL Girotra S Abella B. Trends in survival after in-hospital cardiac arrest during nights and weekends. J Am Coll Cardiol. (2018) 71:402–11. 10.1016/j.jacc.2017.11.043 29389356 PMC5858924

[13] Chan PS Spertus JA Kennedy K Nallamothu BK Starks MA Girotra S. In-hospital cardiac arrest survival in the United States during and after the initial novel coronavirus disease 2019 pandemic surge. Circ Cardiovasc Qual Outcomes. (2022) 15:e008420. 10.1161/CIRCOUTCOMES.121.008420 35098727 PMC8852282

[14] Peberdy MA Kaye W Ornato JP Larkin GL Nadkarni V Mancini ME. Cardiopulmonary resuscitation of adults in the hospital: a report of 14 720 cardiac arrests from the National Registry of Cardiopulmonary Resuscitation. Resuscitation. (2003) 58:297–308. 10.1016/S0300-9572(03)00215-6 12969608

[15] Benjamin EJ Muntner P Alonso A Bittencourt MS Callaway CW Carson AP. Heart disease and stroke statistics-2019 update: a report from the American heart association. Circulation. (2019) 139:e56–e528. 10.1161/CIR.0000000000000659 30700139

[16] Milan M Perman SM. Out of hospital cardiac arrest: a current review of the literature that informed the 2015 American heart association guidelines update. Curr Emerg Hosp Med Rep. (2016) 4:164–71. 10.1007/s40138-016-0118-x 30271683 PMC6159945

[17] Mozaffarian D Benjamin EJ Go AS Arnett DK Blaha MJ Cushman M. Heart disease and stroke statistics-−2015 update: a report from the American heart association. Circulation. (2015) 131:e29–e322. 10.1161/CIR.0000000000000152 25520374

[18] Girotra S van Diepen S Nallamothu BK Carrel M Vellano K Anderson ML. Regional variation in out-of-hospital cardiac arrest survival in the United States. Circulation. (2016) 133:2159–68. 10.1161/CIRCULATIONAHA.115.018175 27081119 PMC4889467

[19] Bray J Howell S Ball S Doan T Bosley E Smith K. The epidemiology of out-of-hospital cardiac arrest in Australia and New Zealand: a binational report from the Australasian resuscitation outcomes consortium (Aus-ROC). Resuscitation. (2022) 172:74–83. 10.1016/j.resuscitation.2022.01.011 35077857

[20] Stiell IG Nichol G Wells G de Maio V Nesbitt L Blackburn J. Health-related quality of life is better for cardiac arrest survivors who received citizen cardiopulmonary resuscitation. Circulation. (2003) 108:1939–44. 10.1161/01.CIR.0000095028.95929.B0 14530198

[21] Kilgannon JH Kirchhoff M Pierce L Aunchman N Trzeciak S Roberts BW. Association between chest compression rates and clinical outcomes following in-hospital cardiac arrest at an academic tertiary hospital. Resuscitation. (2017) 110:154–61. 10.1016/j.resuscitation.2016.09.015 27666168 PMC5167634

[22] Balian S Buckler DG Blewer AL Bhardwaj A Abella BS. Variability in survival and post-cardiac arrest care following successful resuscitation from out-of-hospital cardiac arrest. Resuscitation. (2019) 137:78–86. 10.1016/j.resuscitation.2019.02.004 30771450

[23] Centers for Disease Control and Prevention. Cardiac Arrest Registry to Enhance Survival (CARES) National Summary Report (2013).

[24] Daya MR Schmicker RH Zive DM Rea TD Nichol G Buick JE. Out-of-hospital cardiac arrest survival improving over time: results from the resuscitation outcomes consortium (ROC). Resuscitation. (2015) 91:108–15. 10.1016/j.resuscitation.2015.02.003 25676321 PMC4433591

[25] Yan S Gan Y Jiang N Wang R Chen Y Luo Z. The global survival rate among adult out-of-hospital cardiac arrest patients who received cardiopulmonary resuscitation: a systematic review and meta-analysis. Crit Care. (2020) 24:1–13. 10.1186/s13054-020-2773-2 32087741 PMC7036236

[26] Richardson ASC Schmidt M Bailey M Pellegrino VA Rycus PT Pilcher DV. cardio-pulmonary resuscitation (ECPR), trends in survival from an international multicentre cohort study over 12-years. Resuscitation. (2017) 112:34–40. 10.1016/j.resuscitation.2016.12.009 27993632

[27] Wang G Chen X Qiao L Mei Y Lv J Huang X. Comparison of extracorporeal and conventional cardiopulmonary resuscitation: a meta-analysis of 2 260 patients with cardiac arrest. World J Emerg Med. (2017) 8:5. 10.5847/wjem.j.1920-8642.2017.01.001 28123613 PMC5263037

[28] Twohig CJ Singer B Grier G Finney SJ A. systematic literature review and meta-analysis of the effectiveness of extracorporeal-CPR vs. conventional-CPR for adult patients in cardiac arrest. J Intensivs Care Soc. (2019) 20:347–57. 10.1177/1751143719832162 31695740 PMC6820228

[29] Ouweneel DM Schotborgh JV Limpens J Sjauw KD Engström AE Lagrand WK. Extracorporeal life support during cardiac arrest and cardiogenic shock: a systematic review and meta-analysis. Intensive Care Med. (2016) 42:1922–34. 10.1007/s00134-016-4536-8 27647331 PMC5106498

[30] Chen Z Liu C Huang J Zeng P Lin J Zhu R. Clinical efficacy of extracorporeal cardiopulmonary resuscitation for adults with cardiac arrest: meta-analysis with trial sequential analysis. BioMed Res Int. (2019) 2019:6414673. 10.1155/2019/6414673 31360719 PMC6652040

[31] Ortega-Deballon I Hornby L Shemie SD Bhanji F Guadagno E. Extracorporeal resuscitation for refractory out-of-hospital cardiac arrest in adults: a systematic review of international practices and outcomes. Resuscitation. (2016) 101:12–20. 10.1016/j.resuscitation.2016.01.018 26836946

[32] Beyea MM Tillmann BW Iansavichene AE Randhawa VK van Aarsen K Nagpal AD. Neurologic outcomes after extracorporeal membrane oxygenation assisted CPR for resuscitation of out-of-hospital cardiac arrest patients: a systematic review. Resuscitation. (2018) 130:146–58. 10.1016/j.resuscitation.2018.07.012 30017957

[33] Karve S Lahood D Diehl A Burrell A Tian DH Southwood T. The impact of selection criteria and study design on reported survival outcomes in extracorporeal oxygenation cardiopulmonary resuscitation (ECPR): a systematic review and meta-analysis. Scand J Trauma Resusc Emerg Med. (2021) 29:1–9. 10.1186/s13049-021-00956-5 34565435 PMC8474891

[34] Miraglia D Miguel LA Alonso W. Extracorporeal cardiopulmonary resuscitation for in- and out-of-hospital cardiac arrest: systematic review and meta-analysis of propensity score-matched cohort studies. J Am Coll Emerg Phys Open. (2020) 1:342–61. 10.1002/emp2.12091 33000057 PMC7493557

[35] Downing J Al Falasi R Cardona S Fairchild M Lowie B Chan C. How effective is extracorporeal cardiopulmonary resuscitation (ECPR) for out-of-hospital cardiac arrest? A systematic review and meta-analysis. Am J Emerg Med. (2022) 51:127–38. 10.1016/j.ajem.2021.08.072 34735971

[36] Holmberg MJ Geri G Wiberg S Guerguerian AM Donnino MW Nolan JP. Extracorporeal cardiopulmonary resuscitation for cardiac arrest: a systematic review HHS public access. Resuscitation. (2018) 131:91–100. 10.1016/j.resuscitation.2018.07.029 30063963 PMC6441971

[37] Tonna JE Selzman CH Girotra S Presson AP Thiagarajan RR Becker LB. Patient and institutional characteristics influence the decision to use extracorporeal cardiopulmonary resuscitation for in-hospital cardiac arrest. J Am Heart Assoc. (2020) 9:e015522. 10.1161/JAHA.119.015522 32347147 PMC7428578

[38] Blumenstein J Leick J Liebetrau C Kempfert J Gaede L Groß S. Extracorporeal life support in cardiovascular patients with observed refractory in-hospital cardiac arrest is associated with favourable short and long-term outcomes: a propensity-matched analysis. Eur Heart J Acute Cardiovasc Care. (2016) 5:13–22. 10.1177/2048872615612454 26503919

[39] Cho YH Kim WS Sung K Jeong DS Lee YT Park PW. Management of cardiac arrest caused by acute massive pulmonary thromboembolism: importance of percutaneous cardiopulmonary support. ASAIO J. (2014) 60:280–3. 10.1097/MAT.0000000000000063 24625535

[40] Chou TH Fang CC Yen ZS Lee CC Chen YS Ko WJ. An observational study of extracorporeal CPR for in-hospital cardiac arrest secondary to myocardial infarction. Emerg Med J. (2014) 31:441–7. 10.1136/emermed-2012-202173 24107999

[41] Lin JW Wang MJ Yu HY Wang CH Chang WT Jerng JS. Comparing the survival between extracorporeal rescue and conventional resuscitation in adult in-hospital cardiac arrests: propensity analysis of 3-year data. Resuscitation. (2010) 81:796–803. 10.1016/j.resuscitation.2010.03.002 20413202

[42] Shin TG Choi JH Jo IJ Sim MS Song HG Jeong YK. Extracorporeal cardiopulmonary resuscitation in patients with in-hospital cardiac arrest: a comparison with conventional cardiopulmonary resuscitation. Crit Care Med. (2011) 39:1–7. 10.1097/CCM.0b013e3181feb339 21057309

[43] Shin TG Jo IJ Sim MS Song YB Yang JH Hahn JY. Two-year survival and neurological outcome of in-hospital cardiac arrest patients rescued by extracorporeal cardiopulmonary resuscitation. Int J Cardiol. (2013) 168:3424–30. 10.1016/j.ijcard.2013.04.183 23664696

[44] Chen YS Yu HY Huang SC Lin JW Chi NH Wang CH. Extracorporeal membrane oxygenation support can extend the duration of cardiopulmonary resuscitation. Crit Care Med. (2008) 36:2529–35. 10.1097/CCM.0b013e318183f491 18679121

[45] Park SB Yang JH Park TK Cho YH Sung K Chung CR. Developing a risk prediction model for survival to discharge in cardiac arrest patients who undergo extracorporeal membrane oxygenation. Int J Cardiol. (2014) 177:1031–5. 10.1016/j.ijcard.2014.09.124 25443259

[46] Dennis M McCanny P D'Souza M Forrest P Burns B Lowe DA. Extracorporeal cardiopulmonary resuscitation for refractory cardiac arrest: a multicentre experience. Int J Cardiol. (2017) 231:131–6. 10.1016/j.ijcard.2016.12.003 27986281

[47] Kagawa E Dote K Kato M Sasaki S Nakano Y Kajikawa M. Should we emergently revascularize occluded coronaries for cardiac arrest? Rapid-response extracorporeal membrane oxygenation and intra-arrest percutaneous coronary intervention. Circulation. (2012) 126:1605–13. 10.1161/CIRCULATIONAHA.111.067538 22899771

[48] Stub D Bernard S Pellegrino V Smith K Walker T Sheldrake J. Refractory cardiac arrest treated with mechanical CPR, hypothermia, ECMO and early reperfusion (the CHEER trial). Resuscitation. (2015) 86:88–94. 10.1016/j.resuscitation.2014.09.010 25281189

[49] Ryu JA Cho YH Sung K Choi SH Yang JH Choi JH. Predictors of neurological outcomes after successful extracorporeal cardiopulmonary resuscitation. BMC Anesthesiol. (2015) 15:1–8. 10.1186/s12871-015-0002-3 25774089 PMC4358703

[50] Park BW Seo DC Moon IK Chung JW Bang DW Hyon MS. Pulse pressure as a prognostic marker in patients receiving extracorporeal life support. Resuscitation. (2013) 84:1404–8. 10.1016/j.resuscitation.2013.04.009 23603288

[51] Haneya A Philipp A Diez C Schopka S Bein T Zimmermann M. A 5-year experience with cardiopulmonary resuscitation using extracorporeal life support in non-postcardiotomy patients with cardiac arrest. Resuscitation. (2012) 83:1331–7. 10.1016/j.resuscitation.2012.07.009 22819880

[52] Han SJ Kim HS Choi HH Hong GS Lee WK Lee SH. Predictors of survival following extracorporeal cardiopulmonary resuscitation in patients with acute myocardial infarction-complicated refractory cardiac arrest in the emergency department: a retrospective study. J Cardiothorac Surg. (2015) 10:1–7. 10.1186/s13019-015-0212-2 25889701 PMC4352552

[53] Jung C Janssen K Kaluza M Fuernau G Poerner TC Fritzenwanger M. Outcome predictors in cardiopulmonary resuscitation facilitated by extracorporeal membrane oxygenation. Clin Res Cardiol. (2016) 105:196–205. 10.1007/s00392-015-0906-4 26303097

[54] Lazzeri C Sori A Bernardo P Picariello C Gensini GF Valente S. In-hospital refractory cardiac arrest treated with extracorporeal membrane oxygenation: a tertiary single center experience. Acute Card Care. (2013) 15:47–51. 10.3109/17482941.2013.796385 23915221

[55] Pozzi M Armoiry X Achana F Koffel C Pavlakovic I Lavigne F. Extracorporeal life support for refractory cardiac arrest: a 10-year comparative analysis. Ann Thorac Surg. (2019) 107:809–16. 10.1016/j.athoracsur.2018.09.007 30365965

[56] Peigh G Cavarocchi N Hirose H. Saving life and brain with extracorporeal cardiopulmonary resuscitation: a single-center analysis of in-hospital cardiac arrests. J Thorac Cardiovasc Surg. (2015) 150:1344–9. 10.1016/j.jtcvs.2015.07.061 26383007

[57] Liu Y Cheng YT Chang JC Chao SF Chang BS. Extracorporeal membrane oxygenation to support prolonged conventional cardiopulmonary resuscitation in adults with cardiac arrest from acute myocardial infarction at a very low-volume centre. Interact Cardiovasc Thorac Surg. (2011) 12:389–93. 10.1510/icvts.2010.256388 21172947

[58] Mazzeffi MA Sanchez PG Herr D Krause E Evans CF Rector R. Outcomes of extracorporeal cardiopulmonary resuscitation for refractory cardiac arrest in adult cardiac surgery patients. J Thorac Cardiovasc Surg. (2016) 152:1133–9. 10.1016/j.jtcvs.2016.06.014 27422361

[59] Lee DS Chung CR Jeon K Park CM Suh GY Song Y. Survival after extracorporeal cardiopulmonary resuscitation on weekends in comparison with weekdays. Ann Thorac Surg. (2016) 101:133–40. 10.1016/j.athoracsur.2015.06.077 26431921

[60] Ellouze O Vuillet M Perrot J Grosjean S Missaoui A Aho S. Comparable outcome of out-of-hospital cardiac arrest and in-hospital cardiac arrest treated with extracorporeal life support. Artif Org. (2018) 42:15–21. 10.1111/aor.12992 28877346

[61] Spangenberg T Meincke F Brooks S Frerker C Kreidel F Thielsen T. “Shock and Go?” extracorporeal cardio-pulmonary resuscitation in the golden-hour of ROSC. Catheter Cardiovasc Intervent. (2016) 88:691–6. 10.1002/ccd.26616 27315227

[62] Avalli L Maggioni E Formica F Redaelli G Migliari M Scanziani M. Favourable survival of in-hospital compared to out-of-hospital refractory cardiac arrest patients treated with extracorporeal membrane oxygenation: an Italian tertiary care centre experience. Resuscitation. (2012) 83:579–83. 10.1016/j.resuscitation.2011.10.013 22056265

[63] Bednarczyk JM White CW Ducas RA Golian M Nepomuceno R Hiebert B. Resuscitative extracorporeal membrane oxygenation for in hospital cardiac arrest: a Canadian observational experience. Resuscitation. (2014) 85:1713–9. 10.1016/j.resuscitation.2014.09.026 25449345

[64] Wang CH Chou NK Becker LB Lin JW Yu HY Chi NH. Improved outcome of extracorporeal cardiopulmonary resuscitation for out-of-hospital cardiac arrest: a comparison with that for extracorporeal rescue for in-hospital cardiac arrest. Resuscitation. (2014) 85:1219–24. 10.1016/j.resuscitation.2014.06.022 24992872

[65] Fagnoul D Taccone FS Belhaj A Rondelet B Argacha JF Vincent JL. Extracorporeal life support associated with hypothermia and normoxemia in refractory cardiac arrest. Resuscitation. (2013) 84:1519–24. 10.1016/j.resuscitation.2013.06.016 23816899

[66] Siao FY Chiu CC Chiu CW Chen YC Chen YL Hsieh YK. Managing cardiac arrest with refractory ventricular fibrillation in the emergency department: conventional cardiopulmonary resuscitation vs. extracorporeal cardiopulmonary resuscitation. Resuscitation. (2015) 92:70–6. 10.1016/j.resuscitation.2015.04.016 25936930

[67] Wengenmayer T Rombach S Ramshorn F Biever P Bode C Duerschmied D. Influence of low-flow time on survival after extracorporeal cardiopulmonary resuscitation (eCPR). Crit Care. (2017) 21:1–6. 10.1186/s13054-017-1744-8 28637497 PMC5480193

[68] Tonna JE Selzman CH Girotra S Presson AP Thiagarajan RR Becker LB. Resuscitation using ECPR during in-hospital cardiac arrest (RESCUE-IHCA) mortality prediction score and external validation. JACC Cardiovasc Interv. (2022) 15:237–47. 10.1016/j.jcin.2021.09.032 35033471 PMC8837656

[69] D'Arrigo S Cacciola S Dennis M Jung C Kagawa E Antonelli M. Predictors of favourable outcome after in-hospital cardiac arrest treated with extracorporeal cardiopulmonary resuscitation: a systematic review and meta-analysis. Resuscitation. (2017) 121:62–70. 10.1016/j.resuscitation.2017.10.005 29020604

[70] Agostinucci JM Ruscev M Galinski M Gravelo S Petrovic T Carmeaux C. Out-of-hospital use of an automated chest compression device: facilitating access to extracorporeal life support or non-heart-beating organ procurement. Am J Emerg Med. (2011) 29:1169–72. 10.1016/j.ajem.2010.06.029 20951528

[71] Cesana F Avalli L Garatti L Coppo A Righetti S Calchera I. Effects of extracorporeal cardiopulmonary resuscitation on neurological and cardiac outcome after ischaemic refractory cardiac arrest. Eur Heart J Acute Cardiovasc Care. (2018) 7:432–41. 10.1177/2048872617737041 29064271

[72] Choi DH Kim YJ Ryoo SM Sohn CH Ahn S Seo DW. Extracorporeal cardiopulmonary resuscitation among patients with out-of-hospital cardiac arrest. Clin Exp Emerg Med. (2016) 3:132. 10.15441/ceem.16.145 27752631 PMC5065341

[73] Hase M Tsuchihashi K Fujii N Nishizato K Kokubu N Nara S. Early defibrillation and circulatory support can provide better long-term outcomes through favorable neurological recovery in patients with out-of-hospital cardiac arrest of cardiac origin. Circul J. (2005) 69:1302–7. 10.1253/circj.69.1302 16247202

[74] Kim SJ Jung JS Park JH Park JS Hong YS Lee SW. An optimal transition time to extracorporeal cardiopulmonary resuscitation for predicting good neurological outcome in patients with out-of-hospital cardiac arrest: a propensity-matched study. Crit Care. (2014) 18:1–15. 10.1186/s13054-014-0535-8 25255842 PMC4189722

[75] Lee SH Jung JS Lee KH Kim HJ Son HS Sun K. Comparison of extracorporeal cardiopulmonary resuscitation with conventional cardiopulmonary resuscitation: Is extracorporeal cardiopulmonary resuscitation beneficial? Korean J Thorac Cardiovasc Surg. (2015) 48:318. 10.5090/kjtcs.2015.48.5.318 26509125 PMC4622025

[76] Maekawa K Tanno K Hase M Mori K Asai Y. Extracorporeal cardiopulmonary resuscitation for patients with out-of-hospital cardiac arrest of cardiac origin: a propensity-matched study and predictor analysis. Crit Care Med. (2013) 41:1186–96. 10.1097/CCM.0b013e31827ca4c8 23388518

[77] Poppe M Weiser C Holzer M Sulzgruber P Datler P Keferböck M. The incidence of “load&go” out-of-hospital cardiac arrest candidates for emergency department utilization of emergency extracorporeal life support: a 1-year review. Resuscitation. (2015) 91:131–6. 10.1016/j.resuscitation.2015.03.003 25779007

[78] Sakamoto T Morimura N Nagao K Asai Y Yokota H Nara S. Extracorporeal cardiopulmonary resuscitation vs. conventional cardiopulmonary resuscitation in adults with out-of-hospital cardiac arrest: a prospective observational study. Resuscitation. (2014) 85:762–8. 10.1016/j.resuscitation.2014.01.031 24530251

[79] Schober A Sterz F Herkner H Wallmueller C Weiser C Hubner P. Emergency extracorporeal life support and ongoing resuscitation: a retrospective comparison for refractory out-of-hospital cardiac arrest. Emerg Med J. (2017) 34:277–81. 10.1136/emermed-2015-205232 28213587

[80] Tanno K Itoh Y Takeyama Y Nara S Mori K Asai Y. Utstein style study of cardiopulmonary bypass after cardiac arrest. Am J Emerg Med. (2008) 26:649–54. 10.1016/j.ajem.2007.09.019 18606315

[81] Venturini JM Retzer E Estrada JR Friant J Beiser D Edelson D. Mechanical chest compressions improve rate of return of spontaneous circulation and allow for initiation of percutaneous circulatory support during cardiac arrest in the cardiac catheterization laboratory. Resuscitation. (2017) 115:56–60. 10.1016/j.resuscitation.2017.03.037 28377296

[82] Yannopoulos D Bartos JA Raveendran G Conterato M Frascone RJ Trembley A. Coronary artery disease in patients with out-of-hospital refractory ventricular fibrillation cardiac arrest. J Am Coll Cardiol. (2017) 70:1109–17. 10.1016/j.jacc.2017.06.059 28838358

[83] Lamhaut L Hutin A Puymirat E Jouan J Raphalen JH Jouffroy R. A pre-hospital extracorporeal cardio pulmonary resuscitation (ECPR) strategy for treatment of refractory out hospital cardiac arrest: an observational study and propensity analysis. Resuscitation. (2017) 117:109–17. 10.1016/j.resuscitation.2017.04.014 28414164

[84] Bougouin W Dumas F Lamhaut L Marijon E Carli P Combes A. Extracorporeal cardiopulmonary resuscitation in out-of-hospital cardiac arrest: a registry study. Eur Heart J. (2020) 41:1961–71. 10.1093/eurheartj/ehz753 31670793

[85] Choi DS Kim T Ro YS Ahn KO Lee EJ Hwang SS. Extracorporeal life support and survival after out-of-hospital cardiac arrest in a nationwide registry: a propensity score-matched analysis. Resuscitation. (2016) 99:26–32. 10.1016/j.resuscitation.2015.11.013 26683472

[86] Le Guen M Nicolas-Robin A Carreira S Raux M Leprince P Riou B. Extracorporeal life support following out-of-hospital refractory cardiac arrest. Crit Care. (2011) 15:1–9. 10.1186/cc9976 21244674 PMC3222065

[87] Leick J Liebetrau C Szardien S Fischer-Rasokat U Willmer M van Linden A. Door-to-implantation time of extracorporeal life support systems predicts mortality in patients with out-of-hospital cardiac arrest. Clin Res Cardiol. (2013) 102:661–9. 10.1007/s00392-013-0580-3 23657432

[88] Yannopoulos D Bartos JA Martin C Raveendran G Missov E Conterato M. Minnesota resuscitation consortium's advanced perfusion and reperfusion cardiac life support strategy for out-of-hospital refractory ventricular fibrillation. J Am Heart Assoc. (2016) 5:e003732. 10.1161/JAHA.116.003732 27412906 PMC4937292

[89] Avalli L Mauri T Citerio G Migliari M Coppo A Caresani M. New treatment bundles improve survival in out-of-hospital cardiac arrest patients: a historical comparison. Resuscitation. (2014) 85:1240–4. 10.1016/j.resuscitation.2014.06.014 24973556

[90] Johnson NJ Acker M Hsu CH Desai N Vallabhajosyula P Lazar S. Extracorporeal life support as rescue strategy for out-of-hospital and emergency department cardiac arrest. Resuscitation. (2014) 85:1527–32. 10.1016/j.resuscitation.2014.08.028 25201611

[91] Lamhaut L Tea V Raphalen JH An K Dagron C Jouffroy R. Coronary lesions in refractory out of hospital cardiac arrest (OHCA) treated by extra corporeal pulmonary resuscitation (ECPR). Resuscitation. (2018) 126:154–9. 10.1016/j.resuscitation.2017.12.017 29253646

[92] Djordjevic I Gaisendrees C Adler C Eghbalzadeh K Braumann S Ivanov B. Extracorporeal cardiopulmonary resuscitation for out-of-hospital cardiac arrest: first results and outcomes of a newly established ECPR program in a large population area. Perfusion. (2021) 37:249–56. 10.1177/0267659121995995 33626985

[93] Okada Y Kiguchi T Kitamura T Iwami T. The association between low pH value and unfavorable neurological outcome among the out-of-hospital cardiac arrest patient treated by extra-corporeal CPR: sensitivity analysis. J Intensive Care. (2020) 8:1–9. 10.1186/s40560-020-00470-3 32714555 PMC7374849

[94] Otani T Sawano H Natsukawa T Nakashima T Oku H Gon C. Low-flow time is associated with a favorable neurological outcome in out-of-hospital cardiac arrest patients resuscitated with extracorporeal cardiopulmonary resuscitation. J Crit Care. (2018) 48:15–20. 10.1016/j.jcrc.2018.08.006 30121514

[95] Hilker M Philip A Arlt M Amann M Lunz D Müller T. Pre-hospital cardiopulmonary resuscitation supported by ECMO: a case series of 6 patients. Thorac Cardiovasc Surg. (2013) 61(S 01):P45. 10.1055/s-0032-1332685

[96] Bartos JA Grunau B Carlson C Duval S Ripeckyj A Kalra R. Improved survival with extracorporeal cardiopulmonary resuscitation despite progressive metabolic derangement associated with prolonged resuscitation. Circulation. (2020) 141:877–86. 10.1161/CIRCULATIONAHA.119.042173 31896278 PMC7069385

[97] Nakashima T Noguchi T Tahara Y Nishimura K Ogata S Yasuda S. Patients with refractory out-of-cardiac arrest and sustained ventricular fibrillation as candidates for extracorporeal cardiopulmonary resuscitation: Prospective multi-center observational study. Circul J. (2019) 83:1011–8. 10.1253/circj.CJ-18-1257 30890669

[98] Sawamoto K Bird SB Katayama Y Maekawa K Uemura S Tanno K. Outcome from severe accidental hypothermia with cardiac arrest resuscitated with extracorporeal cardiopulmonary resuscitation. Am J Emerg Med. (2014) 32:320–4. 10.1016/j.ajem.2013.12.023 24468125

[99] Okada Y Kiguchi T Irisawa T Yamada T Yoshiya K Park C. Development and validation of a clinical score to predict neurological outcomes in patients with out-of-hospital cardiac arrest treated with extracorporeal cardiopulmonary resuscitation. JAMA Netw Open. (2020) 3:e2022920. 10.1001/jamanetworkopen.2020.22920 33231635 PMC7686862

[100] Kagawa E Inoue I Kawagoe T Ishihara M Shimatani Y Kurisu S. Assessment of outcomes and differences between in- and out-of-hospital cardiac arrest patients treated with cardiopulmonary resuscitation using extracorporeal life support. Resuscitation. (2010) 81:968–73. 10.1016/j.resuscitation.2010.03.037 20627526

[101] Lunz D Calabrò L Belliato M Contri E Broman LM Scandroglio AM. Extracorporeal membrane oxygenation for refractory cardiac arrest: a retrospective multicenter study. Intensive Care Med. (2020) 46:973–82. 10.1007/s00134-020-05926-6 32052069

[102] Wang J Ma Q Zhang H Liu S Zheng Y. Predictors of survival and neurologic outcome for adults with extracorporeal cardiopulmonary resuscitation: a systemic review and meta-analysis. Medicine. (2018) 97:6283197. 10.1097/MD.0000000000013257 30508912 PMC6283197

[103] Debaty G Babaz V Durand M Gaide-Chevronnay L Fournel E Blancher M. Prognostic factors for extracorporeal cardiopulmonary resuscitation recipients following out-of-hospital refractory cardiac arrest. A systematic review and meta-analysis. Resuscitation. (2017) 112:1–10. 10.1016/j.resuscitation.2016.12.011 28007504

[104] Yukawa T Kashiura M Sugiyama K Tanabe T Hamabe Y. Neurological outcomes and duration from cardiac arrest to the initiation of extracorporeal membrane oxygenation in patients with out-of-hospital cardiac arrest: a retrospective study. Scand J Trauma Resusc Emerg Med. (2017) 25:1–7. 10.1186/s13049-017-0440-7 28915913 PMC5603067

[105] Kippnich M Lotz C Kredel M Schimmer C Weismann D Sommer C. Venoarterielle extrakorporale Membranoxygenierung beim präklinischen Herz-Kreislauf-Stillstand. Anaesthesist. (2015) 64:580–5. 10.1007/s00101-015-0058-y 26194653

[106] Panchal AR Bartos JA Cabañas JG Donnino MW Drennan IR Hirsch KG. Part 3: adult basic and advanced life support: 2020 American heart association guidelines for cardiopulmonary resuscitation and emergency cardiovascular care. Circulation. (2020) 142:S366–468. 10.1161/CIR.0000000000000918 33081529

[107] Topjian AA Raymond TT Atkins D Chan M Duff JP Joyner BL. Part 4: pediatric basic and advanced life support: 2020 American heart association guidelines for cardiopulmonary resuscitation and emergency cardiovascular care. Circulation. (2020) 142:S469–523. 10.1542/peds.2020-038505D 33081526

[108] Soar J Böttiger BW Carli P Couper K Deakin CD Djärv T. European resuscitation council guidelines 2021: adult advanced life support. Resuscitation. (2021) 161:115–51. 10.1016/j.resuscitation.2021.02.010 33773825

[109] van de Voorde P Turner NM Djakow J de Lucas N Martinez-Mejias A Biarent D. European resuscitation council guidelines 2021: paediatric life support. Resuscitation. (2021) 161:327–87. 10.1016/j.resuscitation.2021.02.015 33773830

[110] Soar J Berg KM Andersen LW Böttiger BW Cacciola S Callaway CW. Adult advanced life support: 2020 international consensus on cardiopulmonary resuscitation and emergency cardiovascular care science with treatment recommendations. Resuscitation. (2020) 156:12. 10.1161/CIR.0000000000000893 33099419 PMC7576326

[111] Soar J Maconochie I Wyckoff MH Olasveengen TM Singletary EM Greif R. 2019 international consensus on cardiopulmonary resuscitation and emergency cardiovascular care science with treatment recommendations. Resuscitation. (2019) 145:e826–80. 31734223 10.1016/j.resuscitation.2019.10.016

[112] Soar J MacOnochie I Wyckoff MH Olasveengen TM Singletary EM Greif R. 2019 international consensus on cardiopulmonary resuscitation and emergency cardiovascular care science with treatment recommendations: summary from the basic life support; advanced life support; pediatric life support; neonatal life support; education, implementation, and teams; and first aid task forces. Circulation. (2019) 140:e424–40. 10.1161/CIR.0000000000000734 31722543

[113] Maconochie IK Aickin R Hazinski MF Atkins DL Bingham R Couto TB. Pediatric life support: 2020 international consensus on cardiopulmonary resuscitation and emergency cardiovascular care science with treatment recommendations. Circulation. (2020) 142:S140–84. 10.1542/peds.2020-038505B 33084393

[114] Richardson AC Tonna JE Nanjayya V Nixon P Abrams DC Raman L. Extracorporeal cardiopulmonary resuscitation in adults. Interim guideline consensus statement from the extracorporeal life support organization. ASAIO J. (2021) 67:221. 10.1097/MAT.0000000000001344 33627592 PMC7984716

[115] Guerguerian AM Sano M Todd M Honjo O Alexander P Raman L. Pediatric extracorporeal cardiopulmonary resuscitation ELSO guidelines. ASAIO J. (2021) 67:229–37. 10.1097/MAT.0000000000001345 33627593

[116] Tonna JE Johnson NJ Greenwood J Gaieski DF Shinar Z Bellezo JM. Practice characteristics of emergency department extracorporeal cardiopulmonary resuscitation (eCPR) programs in the United States: the current state of the art of emergency department extracorporeal membrane oxygenation (ED ECMO). Resuscitation. (2016) 107:38–46. 10.1016/j.resuscitation.2016.07.237 27523953 PMC5475402

[117] Neumar RW Nolan JP Adrie C Aibiki M Berg RA Böttiger BW. Post-cardiac arrest syndrome: epidemiology, pathophysiology, treatment, and prognostication a consensus statement from the International Liaison Committee on Resuscitation. Circulation. (2008) 118:2452–83. 10.1161/CIRCULATIONAHA.108.190652 18948368

[118] Bernard SA Gray TW Buist MD Jones BM Silvester W Gutteridge G. Treatment of comatose survivors of out-of-hospital cardiac arrest with induced hypothermia. N Engl J Med. (2002) 346:557–63. 10.1056/NEJMoa003289 11856794

[119] Hypothermia after Cardiac Arrest Study Group. Mild therapeutic hypothermia to improve the neurologic outcome after cardiac arrest. N Engl J Med. (2002) 346:549–56. 10.1056/NEJMoa012689 11856793

[120] Dankiewicz J Cronberg T Lilja G Jakobsen JC Levin H Ullén S. Hypothermia vs. normothermia after out-of-hospital cardiac arrest. N Engl J Med. (2021) 384:2283–94. 10.1056/NEJMoa2100591 34133859

[121] Sandroni C Nolan JP Andersen LW Böttiger BW Cariou A Cronberg T. ERC-ESICM guidelines on temperature control after cardiac arrest in adults. Intensive Care Med. (2022) 48:261–9. 10.1007/s00134-022-06620-5 35089409

[122] Tonna JE Selzman CH Bartos JA Presson AP Ou Z Jo Y. The association of modifiable mechanical ventilation settings, blood gas changes and survival on extracorporeal membrane oxygenation for cardiac arrest. Resuscitation. (2022) 174:53–61. 10.1016/j.resuscitation.2022.03.016 35331803 PMC9050917

[123] Kashiura M Yasuda H Kishihara Y Tominaga K Nishihara M Hiasa K. Association between short-term neurological outcomes and extreme hyperoxia in patients with out-of-hospital cardiac arrest who underwent extracorporeal cardiopulmonary resuscitation: a retrospective observational study from a multicenter registry. BMC Cardiovasc Disord. (2022) 22:163. 10.1186/s12872-022-02598-6 35410132 PMC9003952

[124] Holmberg MJ Wiberg S Ross CE Kleinman M Hoeyer-Nielsen AK Donnino MW. Trends in survival after pediatric in-hospital cardiac arrest in the United States. Circulation. (2019) 140:1398–408. 10.1161/CIRCULATIONAHA.119.041667 31542952 PMC6803102

[125] Girotra S Spertus JA Li Y Berg RA Nadkarni VM Chan PS. Survival trends in pediatric in-hospital cardiac arrests an analysis from get with the guidelines-resuscitation. Circ Cardiovasc Qual Outcomes. (2013) 6:42–9. 10.1161/CIRCOUTCOMES.112.967968 23250980 PMC3555689

[126] Berg RA Sutton RM Reeder RW Berger JT Newth CJ Carcillo JA. Association between diastolic blood pressure during pediatric in-hospital cardiopulmonary resuscitation and survival. Circulation. (2018) 137:1784–95. 10.1161/CIRCULATIONAHA.117.032270 29279413 PMC5916041

[127] Morgan RW Kirschen MP Kilbaugh TJ Sutton RM Topjian AA. Pediatric in-hospital cardiac arrest and cardiopulmonary resuscitation in the United States: a review. JAMA Pediat. (2021) 168:110–8. 10.1001/jamapediatrics.2020.5039 33226408 PMC8787313

[128] Hamzah M Othman HF Almasri M Al-Subu A Lutfi R. Survival outcomes of in-hospital cardiac arrest in pediatric patients in the USA. Eur J Pediatr. (2021) 180:2513–20. 10.1007/s00431-021-04082-3 33899153

[129] Berg RA Nadkarni VM Clark AE Moler F Meert K Harrison RE. Incidence and outcomes of cardiopulmonary resuscitation in PICUs. Crit Care Med. (2016) 44:798. 10.1097/CCM.0000000000001484 26646466 PMC4809365

[130] Virani SS Alonso A Benjamin EJ Bittencourt MS Callaway CW Carson AP. Heart disease and stroke statistics-−2020 update: a report from the American heart association. Circulation. (2020) 141:e139–596. 10.1161/CIR.0000000000000746 31992061

[131] Kitamura T Iwami T Kawamura T Nagao K Tanaka H Nadkarni VM. Conventional and chest-compression-only cardiopulmonary resuscitation by bystanders for children who have out-of-hospital cardiac arrests: a prospective, nationwide, population-based cohort study. Lancet. (2010) 375:1347–54. 10.1016/S0140-6736(10)60064-5 20202679

[132] Conrad SA Rycus PT Dalton H. Extracorporeal life support registry report 2004. ASAIO J. (2005) 51:4–10. 10.1097/01.MAT.0000151922.67540.E9 15745126

[133] Thiagarajan RR Laussen PC Rycus PT Bartlett RH Bratton SL. Extracorporeal membrane oxygenation to aid cardiopulmonary resuscitation in infants and children. Circulation. (2007) 116:1693–700. 10.1161/CIRCULATIONAHA.106.680678 17893278

[134] Farhat A Ling RR Jenks CL Poon WH Yang IX Li X. Outcomes of pediatric extracorporeal cardiopulmonary resuscitation: a systematic review and meta-analysis. Crit Care Med. (2021) 49:682–92. 10.1097/CCM.0000000000004882 33591019

[135] Meert K Slomine BS Silverstein FS Christensen J Ichord R Telford R. One-year cognitive and neurologic outcomes in survivors of paediatric extracorporeal cardiopulmonary resuscitation. Resuscitation. (2019) 139:299–307. 10.1016/j.resuscitation.2019.02.023 30818016 PMC6574085

[136] Moler FW Silverstein FS Holubkov R Slomine BS Christensen JR Nadkarni VM. Therapeutic hypothermia after in-hospital cardiac arrest in children. N Engl J Med. (2017) 376:318–29. 10.1056/nejmoa1610493 28118559 PMC5310766

[137] Lowry AW Morales DLS Graves DE Knudson JD Shamszad P Mott AR. Characterization of extracorporeal membrane oxygenation for pediatric cardiac arrest in the United States: analysis of the kids' inpatient database. Pediatr Cardiol. (2013) 34:1422–30. 10.1007/s00246-013-0666-8 23503928

[138] Barbaro RP Paden ML Guner YS Raman L Ryerson LM Alexander P. Pediatric extracorporeal life support organization registry international report 2016. ASAIO J. (2017) 63:60–7. 10.1097/MAT.0000000000000475 28557863 PMC5626007

[139] Aharon AS Drinkwater DC Churchwell KB Quisling S Reddy VS Taylor M. Extracorporeal membrane oxygenation in children after repair of congenital cardiac lesions. Ann Thorac Surg. (2001) 72:2095–102. 10.1016/S0003-4975(01)03209-X 11789800

[140] Dalton HJ Siewers RD Fuhrman BP del Nido P Thompson AE Shaver MG. Extracorporeal membrane oxygenation for cardiac rescue in children with severe myocardial dysfunction. Crit Care Med. (1993) 21:1020–8. 8319459 10.1097/00003246-199307000-00016

[141] Duncan BW Ibrahim AE Hraska V del Nido P Laussen PC Wessel DL. Use of rapid-deployment extracorporeal membrane oxygenation for the resuscitation of pediatric patients with heart disease after cardiac arrest. J Thorac Cardiovasc Surg. (1998) 116:305–11. 9699584 10.1016/s0022-5223(98)70131-x

[142] Parra DA Totapally BR Zahn E Jacobs J Aldousany A Burke RP. Outcome of cardiopulmonary resuscitation in a pediatric cardiac intensive care unit. Crit Care Med. (2000) 28:3296–300. 10.1097/00003246-200009000-00030 11008995

[143] Thourani VH Kirshbom PM Kanter KR Simsic J Kogon BE Wagoner S. Venoarterial extracorporeal membrane oxygenation (VA-ECMO) in pediatric cardiac support. Ann Thorac Surg. (2006) 82:138–45. 10.1016/j.athoracsur.2006.02.011 16798204

[144] Shah SA Shankar V Churchwell KB Taylor MB Scott BP Bartilson R. Clinical outcomes of 84 children with congenital heart disease managed with extracorporeal membrane oxygenation after cardiac surgery. ASAIO J. (2005) 51:504–7. 10.1097/01.mat.0000171595.67127.74 16322706

[145] Anton-Martin P Moreira A Kang P Green ML. Outcomes of paediatric cardiac patients after 30 min of cardiopulmonary resuscitation prior to extracorporeal support. Cardiol Young. (2020) 30:607–16. 10.1017/S1047951120000591 32228742

[146] Kramer P Mommsen A Miera O Photiadis J Berger F Schmitt KRL. Survival and mid-term neurologic outcome after extracorporeal cardiopulmonary resuscitation in children. Pediatric Crit Care Med. (2020) 21:e316–24. 10.1097/PCC.0000000000002291 32343108

[147] Philip J Burgman C Bavare A Akcan-Arikan A Price JF Adachi I. Nature of the underlying heart disease affects survival in pediatric patients undergoing extracorporeal cardiopulmonary resuscitation. J Thorac Cardiovasc Surg. (2014) 148:2367–72. 10.1016/j.jtcvs.2014.03.023 24787696

[148] Erek E Aydin S Suzan D Yildiz O Altin F Kirat B. Extracorporeal cardiopulmonary resuscitation for refractory cardiac arrest in children after cardiac surgery. Anatol J Cardiol. (2017) 17:328. 10.14744/AnatolJCardiol.2016.6658 28045013 PMC5469114

[149] Sperotto F Saengsin K Danehy A Godsay M Geisser DL Rivkin M. Modeling severe functional impairment or death following ECPR in pediatric cardiac patients: planning for an interventional trial. Resuscitation. (2021) 167:12–21. 10.1016/j.resuscitation.2021.07.041 34389452

[150] Lasa JJ Rogers RS Localio R Shults J Raymond T Gaies M. Extracorporeal cardiopulmonary resuscitation (E-CPR) during pediatric in-hospital cardiopulmonary arrest is associated with improved survival to discharge. Circulation. (2016) 133:165–76. 10.1161/CIRCULATIONAHA.115.016082 26635402 PMC4814337

[151] Raymond TT Cunnyngham CB Thompson MT Thomas JA Dalton HJ Nadkarni VM. Outcomes among neonates, infants, and children after extracorporeal cardiopulmonary resuscitation for refractory in-hospital pediatric cardiac arrest: a report from the national registry of cardiopulmonary resuscitation. Pediatric Crit Care Med. (2010) 11:362–71. 10.1097/PCC.0b013e3181c0141b 19924027

[152] Ortmann L Prodhan P Gossett J Schexnayder S Berg R Nadkarni V. Outcomes after in-hospital cardiac arrest in children with cardiac disease: a report from get with the guidelines-resuscitation. Circulation. (2011) 124:2329–37. 10.1161/CIRCULATIONAHA.110.013466 22025603

[153] Matos RI Watson RS Nadkarni VM Huang HH Berg RA Meaney PA. Duration of cardiopulmonary resuscitation and illness category impact survival and neurologic outcomes for in-hospital pediatric cardiac arrests. Circulation. (2013) 127:442–51. 10.1161/CIRCULATIONAHA.112.125625 23339874

[154] Alten JA Klugman D Raymond TT Cooper DS Donohue JE Zhang W. Epidemiology and outcomes of cardiac arrest in pediatric cardiac ICUs. Pediatric Crit Care Med. (2017) 18:935. 10.1097/PCC.0000000000001273 28737598 PMC5628130

[155] Joffe AR Lequier L Robertson CMT. Pediatric outcomes after extracorporeal membrane oxygenation for cardiac disease and for cardiac arrest: a review. ASAIO J. (2012) 58:297–310. 10.1097/MAT.0b013e31825a21ff 22643323

[156] Morris MC Wernovsky G Nadkarni VM. Survival outcomes after extracorporeal cardiopulmonary resuscitation instituted during active chest compressions following refractory in-hospital pediatric cardiac arrest. Pediatric Crit Care Med. (2004) 5:440–6. 10.1097/01.PCC.0000137356.58150.2E 15329159

[157] Barrett CS Bratton SL Salvin JW Laussen PC Rycus PT Thiagarajan RR. Neurological injury after extracorporeal membrane oxygenation use to aid pediatric cardiopulmonary resuscitation. Pediatric Crit Care Med. (2009) 10:445–51. 10.1097/PCC.0b013e318198bd85 19451851

[158] Bembea MM Ng DK Rizkalla N Rycus P Lasa JJ Dalton H. Outcomes after extracorporeal cardiopulmonary resuscitation of pediatric in-hospital cardiac arrest: a report from the get with the guidelines-resuscitation and the extracorporeal life support organization registries. Crit Care Med. (2019) 47:e278–85. 10.1097/CCM.0000000000003622 30747771

[159] Conrad SJ Bridges BC Kalra Y Pietsch JB Smith AH. Extracorporeal cardiopulmonary resuscitation among patients with structurally normal hearts. ASAIO J. (2017) 63:781–6. 10.1097/MAT.0000000000000568 29084037

[160] Torres-Andres F Fink EL Bell MJ Sharma MS Yablonsky EJ Sanchez-De-Toledo J. Survival and long-term functional outcomes for children with cardiac arrest treated with extracorporeal cardiopulmonary resuscitation. Pediatric Crit Care Med. (2018) 19:451. 10.1097/PCC.0000000000001524 29528976 PMC5935542

[161] Wolf MJ Kanter KR Kirshbom PM Kogon BE Wagoner SF. Extracorporeal cardiopulmonary resuscitation for pediatric cardiac patients. Ann Thorac Surg. (2012) 94:874–80. 10.1016/j.athoracsur.2012.04.040 22698774

[162] Sivarajan V Best D Brizard CP Shekerdemian LS D'Udekem Y Butt W. Duration of resuscitation prior to rescue extracorporeal membrane oxygenation impacts outcome in children with heart disease. Intensive Care Med. (2011) 37:853–60. 10.1007/s00134-011-2168-6 21369812

[163] Shakoor A Pedroso FE Jacobs SE Okochi S Zenilman A Cheung EW. Extracorporeal cardiopulmonary resuscitation (ECPR) in infants and children: a single-center retrospective study. World J Pediatr Congenit Heart Surg. (2019) 10:582–9. 10.1177/2150135119862598 31496406

[164] Garcia Guerra G Zorzela L Robertson CMT Alton GY Joffe AR Moez EK. Survival and neurocognitive outcomes in pediatric extracorporeal-cardiopulmonary resuscitation. Resuscitation. (2015) 96:208–13. 10.1016/j.resuscitation.2015.07.034 26303570

[165] Walter EMD Alexi-Meskishvili V Huebler M Redlin M Boettcher W Weng Y. Rescue extracorporeal membrane oxygenation in children with refractory cardiac arrest. Interact Cardiovasc Thorac Surg. (2011) 12:929–34. 10.1510/icvts.2010.254193 21429870

[166] Burke CR Chan T Brogan TV McMullan DM. Pediatric extracorporeal cardiopulmonary resuscitation during nights and weekends. Resuscitation. (2017) 114:47–52. 10.1016/j.resuscitation.2017.03.001 28263789

[167] Beshish AG Baginski MR Johnson TJ Deatrick BK Barbaro RP Owens GE. Functional status change among children with extracorporeal membrane oxygenation to support cardiopulmonary resuscitation in a pediatric cardiac ICU: a single institution report. Pediatric Crit Care Med. (2018) 19:665–71. 10.1097/PCC.0000000000001555 29659415

[168] Alsoufi B Awan A Manlhiot C Guechef A Al-Halees Z Al-Ahmadi M. Results of rapid-response extracorporeal cardiopulmonary resuscitation in children with refractory cardiac arrest following cardiac surgery. Eur J Cardio-thorac Surg. (2014) 45:268–75. 10.1093/ejcts/ezt319 23818569

[169] Alsoufi B Al-Radi OO Nazer RI Gruenwald C Foreman C Williams WG. Survival outcomes after rescue extracorporeal cardiopulmonary resuscitation in pediatric patients with refractory cardiac arrest. J Thorac Cardiovasc Surg. (2007) 134:952–9. 10.1016/j.jtcvs.2007.05.054 17903513

[170] Prodhan P Fiser RT Dyamenahalli U Gossett J Imamura M Jaquiss RDB. Outcomes after extracorporeal cardiopulmonary resuscitation (ECPR) following refractory pediatric cardiac arrest in the intensive care unit. Resuscitation. (2009) 80:1124–9. 10.1016/j.resuscitation.2009.07.004 19695762 PMC2969175

[171] Kane DA Thiagarajan RR Wypij D Scheurer MA Fynn-Thompson F Emani S. Rapid-response extracorporeal membrane oxygenation to support cardiopulmonary resuscitation in children with cardiac disease. Circulation. (2010) 122(11 SUPPL_1):S241–8. 10.1161/CIRCULATIONAHA.109.928390 20837920

[172] Huang SC Wu ET Wang CC Chen YS Chang CI Chiu IS. Eleven years of experience with extracorporeal cardiopulmonary resuscitation for paediatric patients with in-hospital cardiac arrest. Resuscitation. (2012) 83:710–4. 10.1016/j.resuscitation.2012.01.031 22306256

[173] Melvan JN Davis J Heard M Trivedi JR Wolf M Kanter KR. Factors associated with survival following extracorporeal cardiopulmonary resuscitation in children. World J Pediatr Congenit Heart Surg. (2020) 11:265–74. 10.1177/2150135120902102 32294013

[174] Mattke AC Stocker CF Schibler A Alphonso N Johnson K Karl TR. newly established extracorporeal life support assisted cardiopulmonary resuscitation (ECPR) program can achieve intact neurological outcome in 60 % of children. Intensive Care Med. (2015) 41:2227–8. 10.1007/s00134-015-4036-2 26359167

[175] de Mul A Nguyen DA Doell C Perez MH Cannizzaro V Karam O. Prognostic evaluation of mortality after pediatric resuscitation assisted by extracorporeal life support. J Pediatr Intensive Care. (2019) 08:057–63. 10.1055/s-0038-1667012 31093456 PMC6517051

[176] Kendirli T Özcan S Havan M Baran C Çakici M Arici B. Pediatric extracorporeal cardiopulmonary resuscitation: single-center study. Turk J Med Sci. (2021) 51:1733–7. 10.3906/sag-2002-10 33350296 PMC8569742

[177] Tsukahara K Toida C Muguruma T. Current experience and limitations of extracorporeal cardiopulmonary resuscitation for cardiac arrest in children: a single-center retrospective study. J Intensive Care. (2014) 2:1–5. 10.1186/s40560-014-0068-x 25705425 PMC4336122

[178] Loaec M Himebauch AS Kilbaugh TJ Berg RA Graham K Hanna R. Pediatric cardiopulmonary resuscitation quality during intra-hospital transport. Resuscitation. (2020) 152:123–30. 10.1016/j.resuscitation.2020.05.003 32422246 PMC7321865

[179] Arlt M Philipp A Voelkel S Graf BM Schmid C Hilker M. Out-of-hospital extracorporeal life support for cardiac arrest: a case report. Resuscitation. (2011) 82:1243–5. 10.1016/j.resuscitation.2011.03.022 21536364

[180] Kelly RB Harrison RE. Outcome predictors of pediatric extracorporeal cardiopulmonary resuscitation. Pediatr Cardiol. (2010) 31:626–33. 10.1007/s00246-010-9659-z 20145916 PMC2886903

[181] Polimenakos AC Rizzo V El-Zein CF Ilbawi MN. Post-cardiotomy rescue extracorporeal cardiopulmonary resuscitation in neonates with single ventricle after intractable cardiac arrest: attrition after hospital discharge and predictors of outcome. Pediatr Cardiol. (2017) 38:314–23. 10.1007/s00246-016-1515-3 27885446

[182] Topjian AA Berg RA. Pediatric out-of-hospital cardiac arrest. Circulation. (2012) 125:2374–8. 10.1161/CIRCULATIONAHA.111.071472 22586292

[183] Nguyen DA de Mul A Hoskote AU Cogo P da Cruz EM Erickson S. Factors associated with initiation of extracorporeal cardiopulmonary resuscitation in the pediatric population: an international survey. ASAIO J. (2022) 68:413–8. 10.1097/MAT.0000000000001495 34074851

[184] Topjian AA de Caen A Wainwright MS Abella BS Abend NS Atkins DL. Pediatric post-cardiac arrest care: a scientific statement from the American heart association. Circulation. (2019) 140:e194–233. 10.1161/CIR.0000000000000697 31242751

[185] Duff JP Topjian AA Berg MD Chan M Haskell SE Joyner BL. 2019 American heart association focused update on pediatric advanced life support: an update to the American heart association guidelines for cardiopulmonary resuscitation and emergency cardiovascular care. Circulation. (2019) 140:e904–14. 10.1161/CIR.0000000000000731 31722551

[186] Moler FW Silverstein FS Holubkov R Slomine BS Christensen JR Nadkarni VM. Therapeutic hypothermia after out-of-hospital cardiac arrest in children. N Engl J Med. (2015) 372:1898–908. 10.1056/NEJMoa1411480 25913022 PMC4470472

[187] Bembea MM Nadkarni VM Diener-West M Venugopal V Carey SM Berg RA. Temperature patterns in the early postresuscitation period after pediatric in-hospital cardiac arrest. Pediatric Crit Care Med. (2010) 11:723–30. 10.1097/PCC.0b013e3181dde659 20431503

[188] Topjian AA Telford R Holubkov R Nadkarni VM Berg RA Dean JM. The association of early post-resuscitation hypotension with discharge survival following targeted temperature management for pediatric in-hospital cardiac arrest. Resuscitation. (2019) 141:24–34. 10.1016/j.resuscitation.2019.05.032 31175965 PMC6650337

[189] Lawson S Ellis C Butler K McRobb C Mejak B. Neonatal extracorporeal membrane oxygenation devices, techniques and team roles: 2011 survey results of the United States' extracorporeal life support organization centers. J Extra-Corp Technol. (2011) 43:236. 22416604 PMC4557427

[190] McMullan DM Thiagarajan RR Smith KM Rycus PT Brogan T. Extracorporeal cardiopulmonary resuscitation outcomes in term and premature neonates. Pediatric Crit Care Med. (2014) 15:e9–e16. 10.1097/PCC.0b013e3182a553f3 24141660

[191] Polimenakos AC Wojtyla P Smith PJ Rizzo V Nater M El Zein CF. Post-cardiotomy extracorporeal cardiopulmonary resuscitation in neonates with complex single ventricle: analysis of outcomes. Eur J Cardio-thorac Surg. (2011) 40:1396–405. 10.1016/j.ejcts.2011.01.087 21507672

[192] Laussen PC Guerguerian AM. Establishing and sustaining an ECPR program. Front Pediatrics. (2018) 6:152. 10.3389/fped.2018.00152 29928639 PMC5998755

[193] Su L Spaeder MC Jones MB Sinha P Nath DS Jain PN. Implementation of an extracorporeal cardiopulmonary resuscitation simulation program reduces extracorporeal cardiopulmonary resuscitation times in real patients. Pediatric Crit Care Med. (2014) 15:856–60. 10.1097/PCC.0000000000000234 25162513

[194] Ueda K Yamane T Aguru Y Shibata M. Establishing protocol and simulation-based learning to initiate ECPR in our institution. J Am Coll Cardiol. (2019) 73:2145. 10.1016/S0735-1097(19)32751-2

[195] Stoner AC Schremmer RD Miller MA Davidson KL Pedigo RL Parson JS. Simulation-based system analysis: testing preparedness for extracorporeal membrane oxygenation cannulation in pediatric COVID-19 patients. Pediatr Qual Saf. (2022) 7:e510. 10.1097/pq9.0000000000000510 35071953 PMC8782104

[196] Turek JW Andersen ND Lawson DS Bonadonna D Turley RS Peters MA. Outcomes before and after implementation of a pediatric rapid-response extracorporeal membrane oxygenation program. Ann Thorac Surg. (2013) 95:2140–7. 10.1016/j.athoracsur.2013.01.050 23506632 PMC3747953

[197] Sin SWC Ng PY Ngai WCW Lai PCK Mok AYT Chan RWK. Simulation training for crises during venoarterial extracorporeal membrane oxygenation. J Thorac Dis. (2019) 11:2144. 10.21037/jtd.2019.04.54 31285909 PMC6588776

[198] Sawyer T. Simulation training in extracorporeal cardiopulmonary resuscitation (ECPR). Acad Pediatrics. (2021) 21:438–9. 10.1016/j.acap.2020.11.004 33189906

[199] Sawyer T Burke C McMullan DM Chan T Valdivia H Yalon L. Impacts of a pediatric extracorporeal cardiopulmonary resuscitation (ECPR) simulation training program. Acad Pediatr. (2019) 19:566–71. 10.1016/j.acap.2019.01.005 30684655

[200] di Nardo M David P Stoppa F Lorusso R Raponi M Amodeo A. The introduction of a high-fidelity simulation program for training pediatric critical care personnel reduces the times to manage extracorporeal membrane oxygenation emergencies and improves teamwork. J Thorac Dis. (2018) 10:3409. 10.21037/jtd.2018.05.77 30069336 PMC6051841

[201] Thomas F Chung S Holt DW. Effects of ECMO simulations and protocols on patient safety. J Extra-Corp Technol. (2019) 51:12. 30936583 PMC6436167

[202] Puślecki M Ligowski M Dabrowski M Stefaniak S Ładzińska M Ładziński P. BEST life—“bringing ECMO simulation to life”—how medical simulation improved a regional ECMO program. Artif Org. (2018) 42:1052–61. 10.1111/aor.13332 30043501

[203] Whitmore SP Gunnerson KJ Haft JW Lynch WR VanDyck T Hebert C. Simulation training enables emergency medicine providers to rapidly and safely initiate extracorporeal cardiopulmonary resuscitation (ECPR) in a simulated cardiac arrest scenario. Resuscitation. (2019) 138:68–73. 10.1016/j.resuscitation.2019.03.002 30862530

[204] Weems MF Friedlich PS Nelson LP Rake AJ Klee L Stein JE. The role of extracorporeal membrane oxygenation simulation training at extracorporeal life support organization centers in the United States. Simul Healthcare. (2017) 12:233–9. 10.1097/SIH.0000000000000243 28609315

[205] Lasa JJ Jain P Raymond TT Minard CG Topjian A Nadkarni V. Extracorporeal cardiopulmonary resuscitation in the pediatric cardiac population: in search of a standard of care. Pediatric Crit Care Med. (2018) 19:125. 10.1097/PCC.0000000000001388 29206729 PMC6186525

[206] Ross P Nickson C Sheldrake J McClure J. Using in-situ simulation for extracorporeal cardiopulmonary resuscitation (ECPR) guideline development. SSRN Elect J. (2019) 2019:21. 10.2139/ssrn.3409806

[207] Pang G Futter C Pincus J Dhanani J Laupland KB. Development and testing of a low cost simulation manikin for extracorporeal cardiopulmonary resuscitation (ECPR) using 3-dimensional printing. Resuscitation. (2020) 149:24–9. 10.1016/j.resuscitation.2020.01.032 32045665

