# Supplementary figures and images for: Extracorporeal cardiopulmonary resuscitation in adults and children: A review of literature, published guidelines and pediatric single-center program building experience
Source: Front Med (Lausanne). 2022 Nov 21;9:935424. doi: 10.3389/fmed.2022.935424 (PMC9720280; doi:10.3389/fmed.2022.935424)

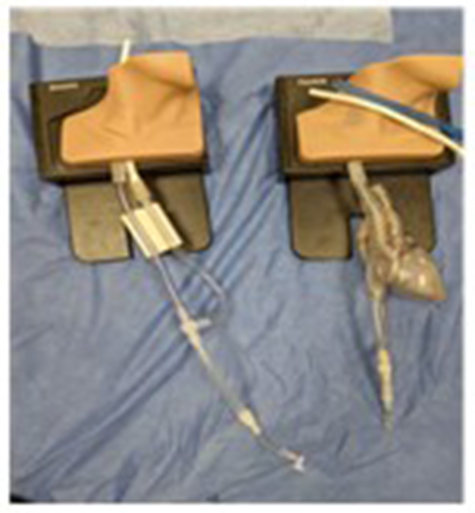

Supplement: Supplementary Image 1 — RediStik™ ECMO Cannulation Trainer. ECMO: extracorporeal membrane oxygenation (https://www.youtube.com/watch?v=UwCJNP94g30&list=PLvWZmgauEkKKcGfI99fYOV9viFyTu03f1&index=2). [file Image_1.JPEG]
